# Self-assembled PROTACs enable glycoproteins degradation in the living cells†

**DOI:** 10.1039/d5sc00400d

**Published:** 2025-04-02

**Authors:** Haoyu Chen, Liu Zang, Pavel Kielkowski

**Affiliations:** a Department of Chemistry, LMU Munich Würmtalstr. 201 81375 Munich Germany pavel.kielkowski@cup.lmu.de

## Abstract

We report here a two-component proteolysis targeting chimeras (PROTACs) strategy selectively targeting *O*-GalNAcylated and *O*-GlcNAcylated proteins for proteasomal degradation, which leads to severe toxicity in human cancer cell lines through perturbation of critical metabolic and signaling pathways governed by glycoproteins. Our approach termed as GlyTAC leverages from metabolic incorporation of easily accessible and cell-permeable peracetylated *N*-acetylglucosamine (GlcNAc) or *N*-acetylgalactosamine (GalNAc) analogues bearing an azido group into glycoproteins. In the living cells, the azido-modified glycoproteins serve as covalent anchors for the introduction of thalidomide moiety by strain-promoted azide–alkyne cycloaddition (SPAAC) to recruit E3 ligase cereblon (CRBN), resulting in stepwise ubiquitination of ‘sensitized’ proteins and their degradation by proteasome. We show the efficiency of the system in a series of human cancer cell lines and verify the mechanistic pathway by performing control experiments at each stage of the process. Given the characteristic features of cancer cells including fast nutrient turnover, and overall increase of protein glycosylation, as well as the low cytotoxicity of the individual components, our approach may open a feasible strategy in cancer therapy.

## Abstract 2

The GlyTAC strategy leverages the metabolic sensitization of glycoproteins to facilitate their conjugation with a proteasomal-targeting group in the second step. This functionalization enables novel gain-of-function post-translational modification.

## Graphical abstract

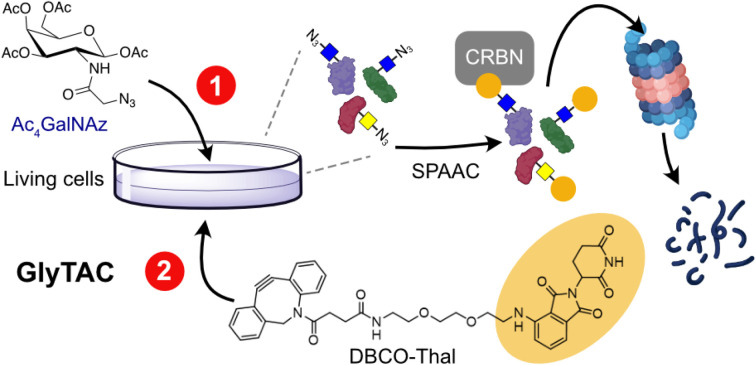

## Introduction

**Fig. 1:**
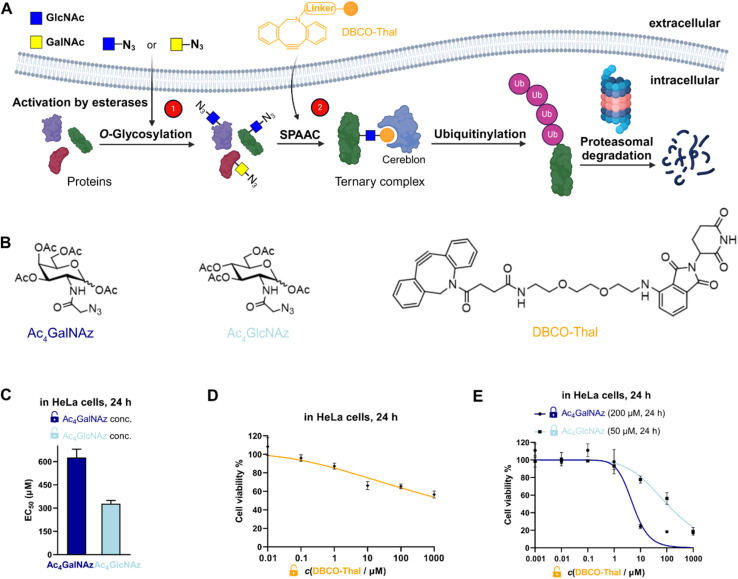
Overview of the GlyTAC strategy. (A) Scheme of the GlyTAC strategy. (B) Structures of Ac_4_GalNAz, Ac_4_GlcNAz and DBCO-thalidomide (DBCO-Thal) used in the study. (C) Bar plot comparing cytotoxicity of GalNAc and GlcNAc analogues in HeLa cells after 24 h incubation, *n* = 3. (D) Concentration-dependent cytotoxicity of DBCO-Thal in HeLa cells after 24 h incubation. Concentration range between 10 nM and 1 mM, *n* = 3. (E) Proof-of-concept experiment of two-component GlyTAC-induced cytotoxicity. DBCO-Thal concentration-dependent cytotoxicity after incubation with Ac_4_GalNAz (200 μM, 24 h) or Ac_4_GlcNAz (50 μM, 24 h) in HeLa cells, *n* = 3. (C–E) Cytotoxicity was determined by MTT cell viability assay; concentrations listed were final concentration of each species in complete cell culture medium.

PROTACs brought new possibilities in the design of novel drug scaffolds and chemical biology tools.^1–3^ Thus far many approaches and alternations of PROTACs have been developed to largely expand their modality.^4^ However, there are only a few examples in which the PROTAC is self-assembled in living cells to induce degradation of a protein of interest (POI). One example is tetrazine ligation-based strategy to bring oncogenic BRD4 or ERK1/2 and cereblon in proximity in the living cells, resulting in their controlled degradation by ubiquitin–proteasome system (UPS).^5^ The advantages lie in improved cell permeability of smaller precursor molecules and optimized versatility of the linker length, which is thought crucial for efficient degradation of a POI by PROTAC approach. PROTACs are typically developed to degrade selectively one protein target. However, the selected POI might be present in cells, tissues, and organisms in different proteoforms, which possess different metabolic or signaling functions resulting in complex responses. More specifically, protein post-translational modifications (PTMs) such as phosphorylation, acetylation and glycosylation significantly extend the number of proteoforms present in the different cell types and may switch POIs' catalytic activities, cellular localization and downstream signaling. Focusing on protein glycosylation, which is fundamental for many signaling pathways in cellular metabolism, there are currently no available platforms for selective degradation of glycoproteins.^6^ Furthermore, protein glycosylation is significantly altered in cancer cells, featured by typically an overexpression of glycosyl-transferases, leading to overall increased glycosylation levels and *de novo* glycosylations.^6–8^ The high metabolic rate of cancer cells requires enhanced nutrient uptake and triggers profound metabolic reprogramming which contributes to the pathophysiology of the disease.^9^ The accelerated turnover of the nutrients in cancer cells can be hijacked for diagnostic purposes, such as using 2-deoxy-2-(fluoro-^18^*F*)-d-glucose as a contrast agent in positron emission tomography (PET).^10–12^ Here, we report the two-component system, in which the first component ‘sensitizes’ specific protein targets in the cells, while the second component prompts selective protein degradation (Fig. 1A). In the first step, the sugar GalNAc or GlcNAc analogues (Fig. 1B) are metabolically introduced as PTMs into glycoproteins, which are subsequently self-assembled through SPAAC with thalidomide analogue to form the ‘active’ PROTAC in the second step (Fig. 1A). The combination of two non-toxic precursors induces proteome-wide changes, degradation of ‘tagged’ glycoproteins and leads inevitably to cell death. The GlyTAC, as we named the strategy, leverages the specific metabolic activity of glycosyl-transferases together with DBCO-thalidomide (DBCO-Thal) to prime cytotoxicity in cancer cell lines.

## Results and discussion

### Synthesis of DBCO-Thal and cytotoxicity testing

To test the validity of two-component GlyTAC strategy, we selected two commonly used and commercially available cell permeable glycosylation analogues Ac_4_GalNAz and Ac_4_GlcNAz (Fig. 1B). These two probes were proved to be well-accepted by corresponding enzymatic writers and efficiently incorporated into glycoproteins.^13–16^ Next, we have designed and synthesized the second component containing dibenzocyclooctyne (DBCO) conjugated with thalidomide to facilitate copper-free ‘click’ reaction and thus to assemble active PROTAC for CRBN recruitment (Fig. 1B).^17,18^ The DBCO and thalidomide are linked through short ethylene glycol units to improve the overall solubility and leave necessary spatial distance, which allows the formation of ternary complex between thalidomide moiety and CRBN. The DBCO-Thal was synthesized sequentially by nucleophilic aromatic substitution (S_N_Ar) on 4-fluoro-thalidomide, followed by Boc-deprotection to release primary amine of the linker, and finally amide bond formation with readily available DBCO-NHS ester (Fig. S1†). The overall isolated yield was above 64%. The stability of DBCO-Thal was tested in water for 2 h – 5 days without observable decomposition (Fig. S2†). Next, MTT cell viability assay was used to evaluate the cytotoxicity of individual GlyTAC components including Ac_4_GalNAz, Ac_4_GlcNAz and DBCO-Thal. After 24 h treatment in HeLa cells, the Ac_4_GalNAz gave EC_50_ value at 625 μM and Ac_4_GlcNAz at 328 μM, with no cytotoxicity up to 300 μM and 150 μM, respectively (Fig. 1C and S3†). The DBCO-Thal was not cytotoxic up to 1 μM and showed only minor cytotoxicity up to 1 mM in cell culture medium (Fig. 1D). Analogous cell viability assays were carried out in Chinese hamster ovary (CHO) cells, providing comparable results and corroborating low toxicity of all components (Fig. S4†). The metabolic incorporation efficiency of Ac_4_GalNAz was tested by in-gel fluorescence analysis after SPAAC with DBCO-TAMRA (Fig. S5†). Together, the cell viability assays showed wide concentration tolerance available for the application of proposed two-component system and hence the feasibility of GlyTAC approach.

### GlyTAC proof-of-principle

Given the complexity and dynamics of protein glycosylation, we next tested if the GlyTAC self-assembly *via* SPAAC would be sufficient to induce proteasomal degradation of glycoproteins. The degradation of glycoproteins in turn may induce cytotoxicity due to broad dysregulation of corresponding metabolic and signaling pathways. In the proof-of-principle experiment, HeLa cells were firstly incubated with Ac_4_GalNAz at highest non-toxic concentration (200 μM) for 24 h to reach saturated portions of azido-containing proteins, then the cell culture medium was exchanged with fresh medium containing increasing concentrations of DBCO-Thal. As shown in Fig. 1E, such GlyTAC approach displayed a rapid increase in cytotoxicity above 1 μM of DBCO-Thal and EC_50_ at 5 μM (Fig. 1E). The parallel experiment with 50 μM Ac_4_GlcNAz treatment in the first step led to significantly higher EC_50_ of DBCO-Thal (approx. 100 μM), shown in a similar concentration-dependent but flattened cytotoxicity curve when compared with Ac_4_GalNAz (Fig. 1E). Together, the strong increase in cytotoxicity of DBCO-Thal after pre-treatment with Ac_4_GalNAz demonstrated promising functionality of GlyTAC approach.

### Characterization of the GlyTAC system

**Fig. 2:**
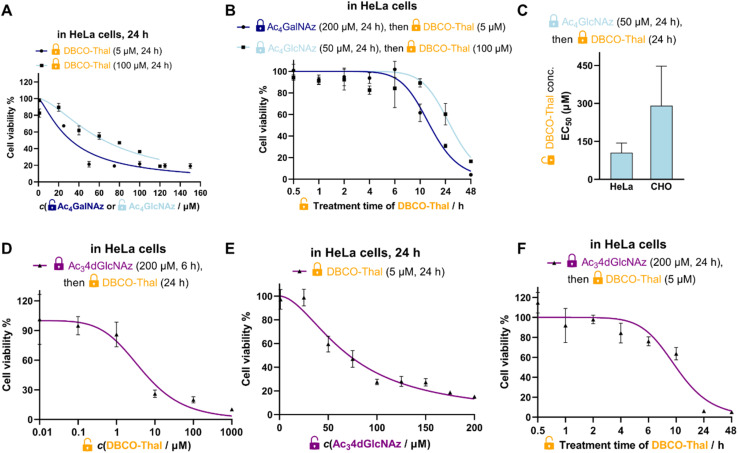
Characterization and optimization of GlyTAC system. (A) Ac_4_GalNAz and Ac_4_GlcNAz concentration-dependency with fixed treatment conditions of DBCO-Thal in HeLa cells, *n* = 3. (B) DBCO-Thal time-dependency in HeLa cells, *n* = 3. (C) Cell type-dependency of the GlyTACs with fixed treatment conditions of Ac_4_GlcNAz in HeLa or CHO cells, *n* = 3. (D) DBCO-Thal concentration-dependency with fixed treatment conditions of Ac_3_4dGlcNAz in HeLa cells, *n* = 3, (E) Ac_3_4dGlcNAz concentration-dependency with fixed treatment conditions of DBCO-Thal in HeLa cells, *n* = 3, (F) time-dependency of DBCO-Thal with fixed treatment conditions of Ac_3_4dGlcNAz in HeLa cells, *n* = 3. (A–F) The graph symbols are mean values and error bars represent standard deviations (SD).

To better understand, characterize and optimize the dynamic range of our two-component system, we tested the concentration- and time-dependences of both glycosylation analogues and DBCO-Thal. First, at the fixed EC_50_ concentration and incubation time of DBCO-Thal, Ac_4_GalNAz and Ac_4_GlcNAz were titrated in HeLa cells respectively to show higher efficiency of Ac_4_GalNAz precursor (Fig. 2A). While EC_50_ was achieved with Ac_4_GalNAz at 30 μM when coupled with 5 μM DBCO-Thal, Ac_4_GlcNAz showed lower toxicity at the same concentration. Second, the treatment time of DBCO-Thal was optimized with Ac_4_GalNAz and Ac_4_GlcNAz pre-incubation to display a major difference between two glycosylation analogues, requiring 14.5 h and 27 h treatment to reach EC_50_ (Fig. 2B), respectively. To test the persistence of the GlyTAC approach, the HeLa cells were firstly incubated with 200 μM Ac_4_GalNAz and subsequently with different concentrations of DBCO-Thal for 6 h, after which the cell culture medium was exchanged for fresh medium without DBCO-Thal. Further incubation of the cells for 24 and 48 h did not lead to cells recovery after 5 μM DBCO-Thal treatment, and only partial recovery after 2.5 μM DBCO-Thal treatment (Fig. S6†). In contrast, the prolonged incubation time with DBCO-Thal for two and four days did not increase the cytotoxicity (Fig. S7†). Next, we evaluated the cell type-dependent cytotoxicity of DBCO-Thal in Ac_4_GlcNAz pre-treated HeLa and CHO cells, observing around two-fold lower EC_50_ in HeLa cells, which can be explained by lower glycosylation levels in CHO cells (Fig. 2C).^19^ Since many glycosylated proteins are located at plasma membrane with a glycan oriented towards extracellular space, we have considered to utilize a 4-deoxy GlcNAz analogue (Ac_3_4dGlcNAz), which is mainly incorporated into intracellular proteins as the vacant C-4 hydroxy group restricts the prolongation of glycosylation chain (Fig. 2D–F).^20^

Although Ac_3_4dGlcNAz analogue showed promising efficacy, it was not enhancing active enough to counterweight its synthetic accessibility. In comparison with synthetic routes to obtain Ac_4_GalNAz (4 steps, Fig. S8†) and Ac_4_GlcNAz (1 step) from cheap starting building blocks (∼85 USD for 5 g), the published Ac_3_4dGlcNAz synthetic route required 11 steps with rather low overall yield.^20,21^ Taken together, pre-treatment of Ac_4_GalNAz at 200 μM for 24 h followed by conjugation with DBCO-Thal at 5 μM for 24 h indicated the most promising cytotoxicity effect in combination with the best accessibility.

### GlyTACs lead to proteome-wide ubiquitination and Nup62 degradation

**Fig. 3:**
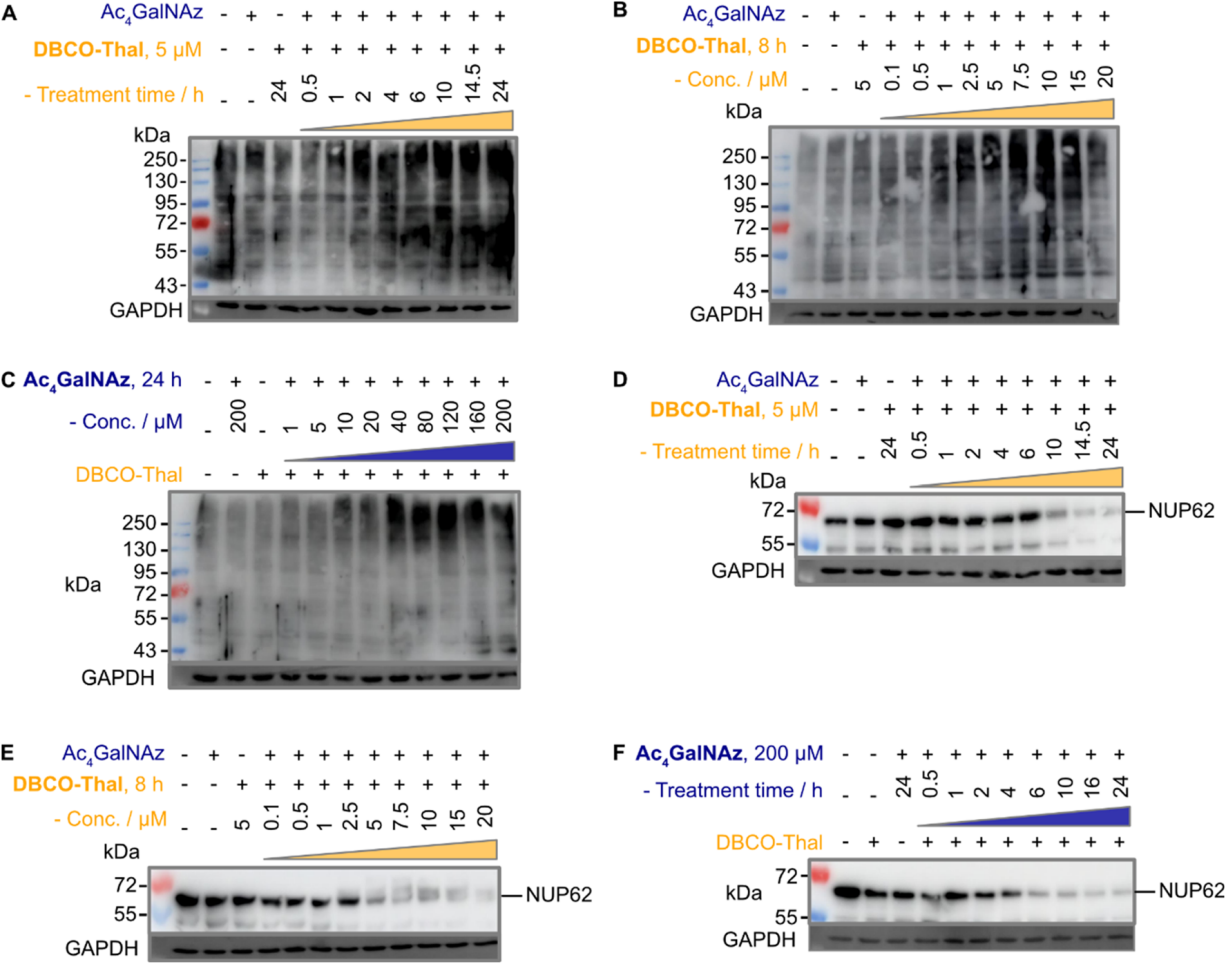
GlyTACs increase protein ubiquitination and Nup62 degradation. (A–C) Western blots using anti-polyubiquitin antibody of (A) DBCO-Thal time-dependent protein ubiquitination, Ac_4_GalNAz was constant at 200 μM for 24 hours, (B) DBCO-Thal concentration-dependent protein ubiquitination, Ac_4_GalNAz was constant at 200 μM for 24 hours and (C) Ac_4_GalNAz concentration-dependent protein ubiquitination, DBCO-Thal was constant at 5 μM for 10 hours. (D–F) Western blot using anti-Nup62 antibody of (D) DBCO-Thal time-dependent, Ac_4_GalNAz was constant at 200 μM for 24 hours, (E) DBCO-Thal concentration-dependent, Ac_4_GalNAz was constant at 200 μM for 24 hours and (F) Ac_4_GalNAz time-dependent, DBCO-Thal was constant at 5 μM for 16 hours.

The anticipated mechanism of action is that the glycoproteins are first modified with azide-containing GalNAc analogues, which are subsequently conjugated *via* SPAAC with thalidomide to recruit CRBN and trigger downstream ubiquitination. The critical step leading to efficient degradation of tagged proteins is therefore adequate ubiquitination. Given the predicted proteome-wide modification of glycoproteins with thalidomide in proximity, we expected a global increase in ubiquitination. Indeed, immunoblotting with anti-polyubiquitin antibody showed marked ubiquitination increases in concentration- and time-dependent manner of both components (Fig. 3A–C). Next, to validate our approach, we selected well-described nuclear pore glycoprotein p62 (Nup62) as immunoblotting target, which is heavily *O*-GlcNAcylated.^22^ As expected, Nup62 demonstrated a significant decrease in a similar concentration- and time-dependent manner when subjected to two-component GlyTACs treatment (Fig. 3D–F). This could be explained by C-4 hydroxyl group epimerization of Ac_4_GalNAz. The 4′-epimerase GALE efficiently transforms UDP (uridine diphosphate)-GalNAz substrate to UDP-GlcNAz, which is accepted by *O*-GlcNAc transferase (OGT) to initiate protein *O*-GlcNAcylation.^23^ Gratifyingly, there was no observable change in Nup62 levels when treated solely with the Ac_4_GalNAz or DBCO-Thal (Fig. 3D–F). Together, the significant Nup62 degradation and global increase in ubiquitination validated GlyTACs mechanism of action based on protein glycosylation and CRBN recruitment.

### GlyTACs mechanism of action

**Fig. 4:**
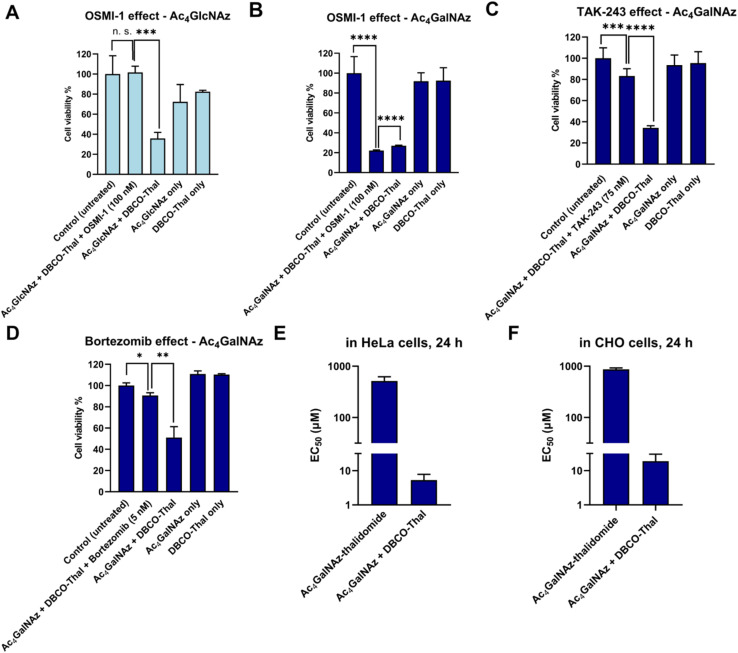
GlyTACs mechanism of action. (A) OSMI-1 induced *O*-GlcNAcylation inhibition rescued GlyTAC-triggered cell death. (B) OSMI-1 did not rescue *O*-GalNAcylation-induced cell death. (C) Inhibition of ubiquitin-like modifier-activating enzyme 1 by TAK-243 rescues the GlyTAC activity. (D) Proteasome inhibition by bortezomib rescued GlyTAC-triggered cell death. (E and F) The sequential treatment of two components was required to induce GlyTAC-triggered cell death as the cells treated with Ac_4_GalNAz-thalidomide conjugate showed negligible cytotoxicity in HeLa (E) and CHO (F) cells. The data are represented as mean values with standard deviations (*n* = 3 for A, B and D–F, *n* = 6 for C). A *p*-value of <0.05 is considered to indicate a statistically significant difference and marked with * (*p* ≤ 0.05), ** (*p* ≤ 0.01), *** (*p* ≤ 0.001) and **** (*p* ≤ 0.0001); while n. s. indicates not significant statistically.

To further investigate the GlyTACs mechanism of action, we functionally probed each step of the process. The first critical step in GlyTAC system is the incorporation of Ac_4_GalNAz or Ac_4_GlcNAz into glycoproteins. In the case of Ac_4_GlcNAz, the incorporation is catalyzed by OGT, which can be inhibited by a small molecule inhibitor OSMI-1.^24^ To test whether the OSMI-1 abolishes GlyTAC cytotoxicity, HeLa cells were pretreated with the inhibitor before addition of Ac_4_GlcNAz. As expected, subsequent treatment with DBCO-Thal did not result in cytotoxic activity due to OGT inhibition (Fig. 4A). In contrast, OSMI-1 treatment had no impact on Ac_4_GalNAz-based GlyTAC as the *O*-GalNAcylation is not catalyzed by OGT, but about 14 *O*-GalNAc transferases (Fig. 4B). Of note, both metabolic chemical reporters Ac_4_GlcNAz and Ac_4_GalNAz are incorporated into large and diverse groups of glycoproteins. The OSMI-1 inhibition of Ac_4_GlcNAz incorporation demonstrates that the GlcNAz is predominantly incorporated by OGT into *O*-GlcNAcylation sites. On the other hand, the epimerization of GalNAc analogue occurs to some extent, as we showed for Nup62 degradation, but it is not providing enough *O*-GlcNAcylated proteins to retard cytotoxicity with OSMI-1 treatment.

After SPAAC between azido-modified glycoproteins with DBCO-Thal, the next step in the GlyTAC strategy is the recruitment of CRBN to form a ternary complex. The interaction should be in principle outcompeted by the pretreatment with thalidomide. The parallel incubation of Ac_4_GalNAz-treated cells with DBCO-Thal and additional 5 μM thalidomide led to partial rescue of GlyTAC cytotoxicity (Fig. S9†). However, thalidomide competition only functioned within a limited time range (approx. 8–10 h), as the cytotoxicity after standard treatment time of DBCO-Thal (24 h) has always brought significant cytotoxicity. Next, the ubiquitination is necessary for targeting selected proteins to UPS. To test whether the inhibition of ubiquitination process hampers the GlyTAC activity, we used a small compound inhibitor TAK-243 to inhibit the ubiquitin-like modifier-activating enzyme 1 (UBA1).^25^ Cells treatment with GlyTAC compounds and TAK-243 demonstrated that UBA1 activity is necessary for proper GlyTAC function (Fig. 4C). After the CRBN recruitment and subsequent ubiquitination to glycoproteins, they are targeted for degradation by UPS. The proteasomal protein degradation can be inhibited by bortezomib, an anti-cancer small molecule drug.^26,27^ To probe that the proteasomal activity is responsible for protein degradation in the GlyTAC system, we used 5 nM bortezomib to rescue the cytotoxicity of Ac_4_GalNAz-based GlyTAC (Fig. 4D). Next, to corroborate the effects observed using the cytotoxicity assays, the western blot of Nup62 was done for each condition showing the consistency between cytotoxicity assay and mechanism (Fig. S10†). Furthermore, in principle, it might be possible to directly treat the cells with a Ac_4_GalNAc-thalidomide conjugate to achieve the GlyTAC cytotoxicity. Therefore, we synthesized the Ac_4_GalNAz-thalidomide linked through a triazole ring (Fig. S11†) and used it for cell treatment, which resulted in virtually no cytotoxicity in both HeLa and CHO cells (Fig. 4E and F). In general, the Ac_4_GalNAc-thalidomide conjugate may undergo β-elimination and subsequent addition reaction on protein cysteine to introduce chemical *S*-glyco-modification, resulting in its cytotoxicity. However, due to the lack of observed cytotoxicity, we concluded that the eventual *S*-glyco-modification is not sufficient for expressing the overall activity of GlyTAC approach.^28–30^ Moreover, reanalysis of mass spectra acquired by Woo *et al.* from Ac_4_GalNAz-treated cells and glycopeptides enriched by acid-cleavable biotin linker determines the ratio of 4.5 : 1 between metabolic and chemical labelling (approximately 18%, Fig. S12 and Table S1†).^31^ That further corroborates the notion of prevailing metabolic labelling using Ac_4_GalNAz, which was demonstrated in biochemical experiments using sodium hydroxide and protein glycosidases.^30^ Taken together, the functional manipulation of enzymatic writer OGT, thalidomide competition for CRBN, UBA1 inhibition by TAK-243 and proteasome inhibition by bortezomib provide insight into GlyTAC mechanism of action. The comparison between the efficacy of Ac_4_GalNAz-thalidomide (single PROTAC compound) and sequential treatment in cells shows advantage of the two-component GlyTAC approach.

### GlyTAC system triggers proteome-wide metabolic changes

**Fig. 5:**
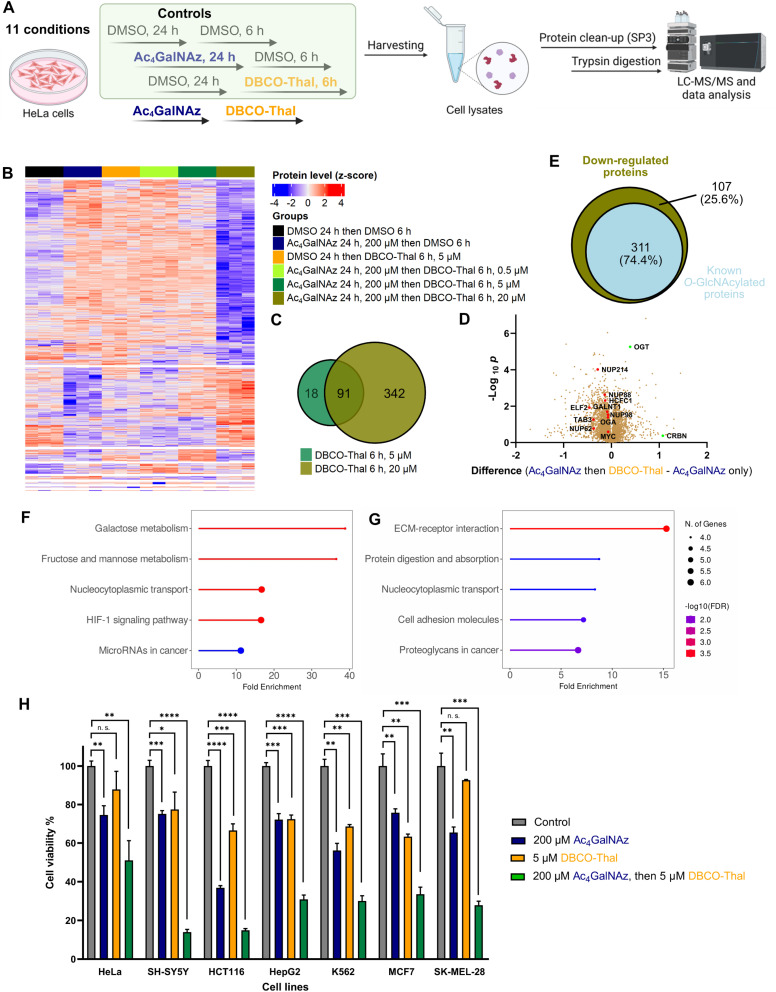
GlyTACs induced whole proteome changes and cancer cell screening. (A) Experimental design of proteomics analysis. (B) Heatmap visualizing up- and down-regulated proteins in all conditions using ANOVA-significant test. (C) Venn diagram showing the overlap of down-regulated proteins in 5 μM and 20 μM DBCO-Thal treatment. (D) Volcano plot comparing GlyTAC (200 μM Ac_4_GalNAz then 5 μM DBCO-Thal) against separate Ac_4_GalNAz treatment as a control, *n* = 3. (E) Venn diagram showing the percentage of known *O*-GlcNAcylated protein contained in significantly down-regulated proteins after GlyTAC treatment. (F and G) KEGG pathway analysis of significantly up-regulated (F) and down-regulated (G) proteins. (H) Screening of 7 human cancer lines after GlyTAC treatment; Ac_4_GalNAz (200 μM) treatment was done for 24 h followed by DBCO-Thal (5 μM) for 16 h. The data are represented as mean values with standard deviations. A *p*-value of <0.05 is considered to indicate a statistically significant difference and marked with * (*p* ≤ 0.05), ** (*p* ≤ 0.01), *** (*p* ≤ 0.001) and **** (*p* ≤ 0.0001); while n. s. indicates not significant statistically.

The broad incorporation of Ac_4_GalNAz into glycoproteins induces significant protein degradation in living cells after DBCO-Thal addition. To characterize changes on the whole proteome level triggered by GlyTACs, a series of analyses were performed using quantitative mass spectrometry-based proteomics (Fig. 5A and Table S2†).^32–36^ In the control experiments where Ac_4_GalNAz or DBCO-Thal were separately used for cell treatment, we observed only minor proteome changes. However, the application of both components with increasing concentration of DBCO-Thal showed anticipated concentration-dependent protein down-regulation (Fig. 5B). Further analysis revealed that from a total of 7187 identified proteins, 109 proteins were down-regulated after 5 μM DBCO-Thal treatment (Fig. 5C). Increasing DBCO-Thal concentration to 20 μM led to more pronounced protein down-regulation of 433 proteins (Fig. 5C). Comparison of both showed an overlap of 91 proteins (Fig. 5C). The proteomics also confirmed down-regulation of Nup62 and three other nuclear pore proteins including Nup214, Nup88 and Nup98 (Fig. 5D). To find how many down-regulated proteins were known to be *O*-GlcNAcylated, which may result from GALE epimerase activity during Ac_4_GalNAz treatment, we compared the list of the down-regulated proteins with *O*-GlcNAc database (Fig. 5E). The search revealed overall down-regulation of 311 known *O*-GlcNAcylated proteins with 20 μM DBCO-Thal treatment (Fig. 5E). The KEGG pathway analysis mainly showed up-regulated proteins associated with carbohydrates metabolism and HIF-1 signaling pathways, suggesting that the dysregulation of glycoproteins has deepened hypoxic stress (Fig. 5F and G). On the other hand, we observed up-regulation of a small portion of 14 proteins including OGT and CRBN (Fig. 5D). Taken together, the proteomics analysis suggests major downregulation of glycoproteins.

### GlyTACs activity screening in cancer cell lines

To better understand susceptibility of different cancer cell lines to GlyTAC treatment, seven human cancer cell lines were screened using fixed concentrations of Ac_4_GalNAz (200 μM) and DBCO-Thal (5 μM, Fig. 5H). The cell lines included HeLa, SH-SY5Y, HCT116, HepG2, K562, MCF7 and SK-MEL-28. Overall, in all tested cancer cell lines, significant cytotoxicity of two-component GlyTACs was observed in comparison to blank control and treatments with Ac_4_GalNAz or DBCO-Thal separately (Fig. 5H). The most pronounced effect was determined in neuroblastoma cells (SH-SY5Y). In other cell lines, relatively high concentration of Ac_4_GalNAz at 200 μM showed somewhat increased cytotoxicity, while DBCO-Thal remained in the same or slightly lower cytotoxicity range. The treatment window might be further extended in stressed or hypoxic cells with increased proteasome activity.^37^ Optimization of treatment conditions in each cancer cell line would likely improve the efficiency of GlyTACs. The selectivity of GlyTAC approach in 2D cell culture is difficult to conduct as the sugar analogues accessibility from a cell culture medium and cell permeability is very high, and hence not representing the tissue or organ environment. Evaluation of the GlyTACs in mice is planned in our laboratory. Together, the presented testing of the GlyTAC strategy in different human cancer cell lines demonstrates feasibility of the approach.

## Conclusions

We have established the two-component GlyTAC approach, which induces severe cytotoxicity in human cancer cell lines while the treatment with each component separately shows only minor effect. The system takes advantage of glycoproteins accessibility *via* metabolic incorporation of PTM mimics containing an azido group, which is used in the second step to covalently anchor DBCO-Thal through SPAAC and subsequently leads to proteasomal degradation of such proteins. Although the covalent bonds between the degrader moiety and POIs prevent the GlyTACs from proceeding catalytically like other PROTAC modalities, we concluded that low cytotoxicity of the individual components and sequential treatment overcomes this problem. More importantly, GlyTACs largely improve the shortcomings of single PROTAC molecules, especially poor aqueous solubility and cell permeability. The efficiency of GlyTACs stems from wide-spread incorporation of azido group into glycoproteins and their self-assembly with thalidomide moiety by SPAAC. Protein PTMs enable to regulate specific subset of the total protein pool, often in cell-type dependent manner, providing thus far unused lever to manipulate physiological process. Further understanding of substrate selectivity of PTM writers is needed to target selected proteins. Ongoing studies in our laboratory are testing this idea.

## Data availability

ESI contains ESI figures, detail experimental procedures, structure characterisations and materials.† Proteomics data are available *via* ProteomeXchange with identifier PXD059044.

## Author contributions

H. C. and P. K. conceptualized the study. H. C. and L. Z. performed all the experiments. P. K. and H. C. wrote and reviewed the manuscript.

## Conflicts of interest

The authors declare no conflict of interest.

## Supplementary Material

SC-016-D5SC00400D-s001

SC-016-D5SC00400D-s002
