# Supplementary material for: Self-assembled PROTACs enable glycoproteins degradation in the living cells
Source: Chem Sci. 2025 Apr 2;16(18):8060–8. doi: 10.1039/d5sc00400d (PMC11976659; doi:10.1039/d5sc00400d)
Supplement: SC-016-D5SC00400D-s001 [file SC-016-D5SC00400D-s001.pdf]

Supplementary Information (SI) for Chemical Science.
This journal is © The Royal Society of Chemistry 2025

Supporting Information

Self-Assembled PROTACs Enable Glycoproteins Degradation in the Living Cells

Haoyu Chen[1], Liu Zang[1], Pavel Kielkowski[1]*

[1] Department of Chemistry, LMU Munich, Würmtalstr. 201, 81375, Munich, Germany.

* Correspondence to pavel.kielkowski@cup.lmu.de

Table of Contents

Supplementary Figures 3

Methods 11

Organic synthesis 11

Cell culture 14

Treatments of two-components GlyTACs in living cells 15

Harvesting HeLa cells 15

Lysates preparation 15

Protein concentration measurement 15

Western blot 16

MTT cytotoxicity assay 17

Cell lysates labelling by DBCO-TAMRA (Figure S5) 19

In-gel fluorescence analysis 20

Direct injection for high-resolution MS1 analysis 20

Cell lysates clean-up with automated SP3 protocol 20

LC-MS/MS measurement 21

Data-independent acquisition (DIA) 21

Computation of measured mass spectra using DIA-NN 21

Data visualization – heatmap (Figure 5B) 22

Data visualization – volcano plot (Figure 5D) 22

Data visualization – Venn diagrams (Figure 5C and 5E) 22

Data visualization – KEGG pathway analysis (Figure 5F and 5G) 23

Uncropped images of western blot 233

NMR spectra 24

References 29

Supplementary Figures

A

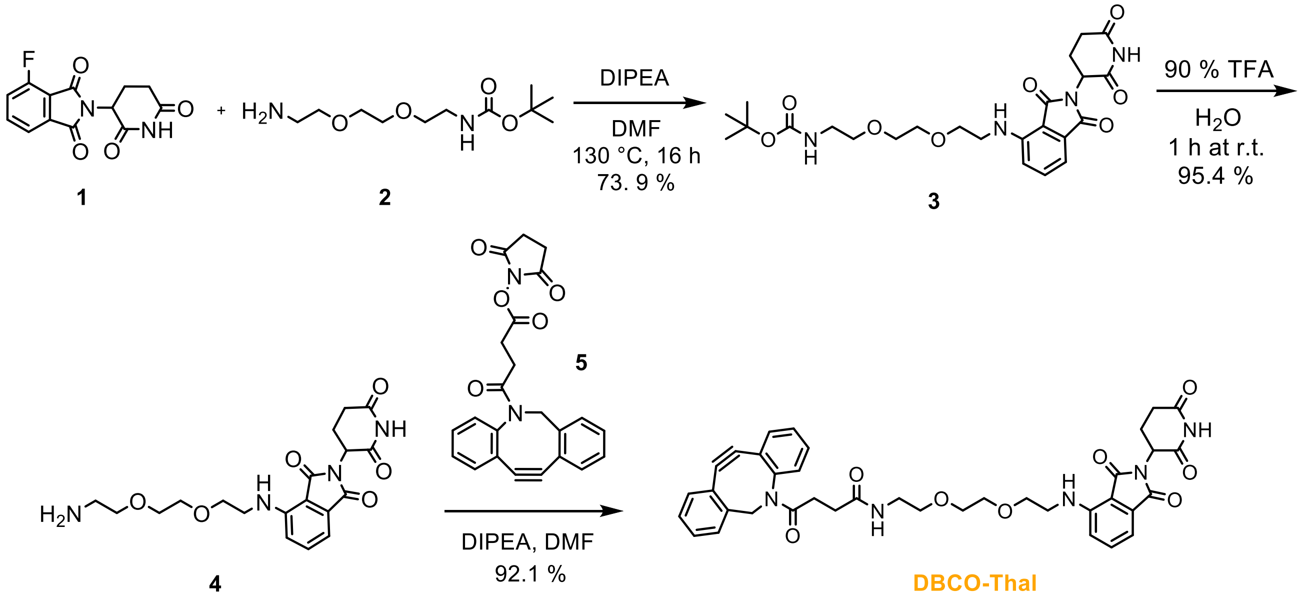

B

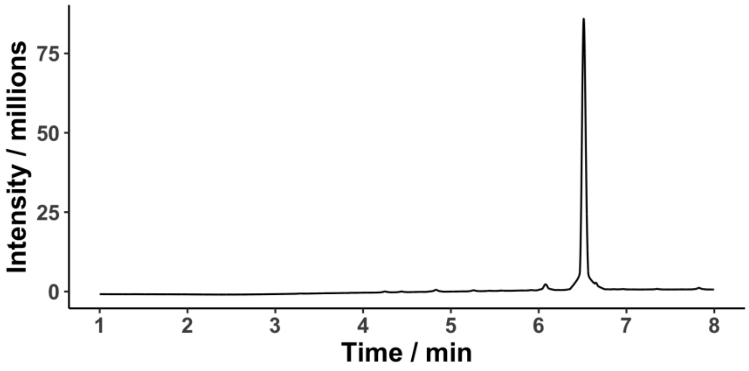

Figure S1. A) Synthesis of DBCO-Thal. **B**) LC-MS chromatogram of DBCO-Thal.

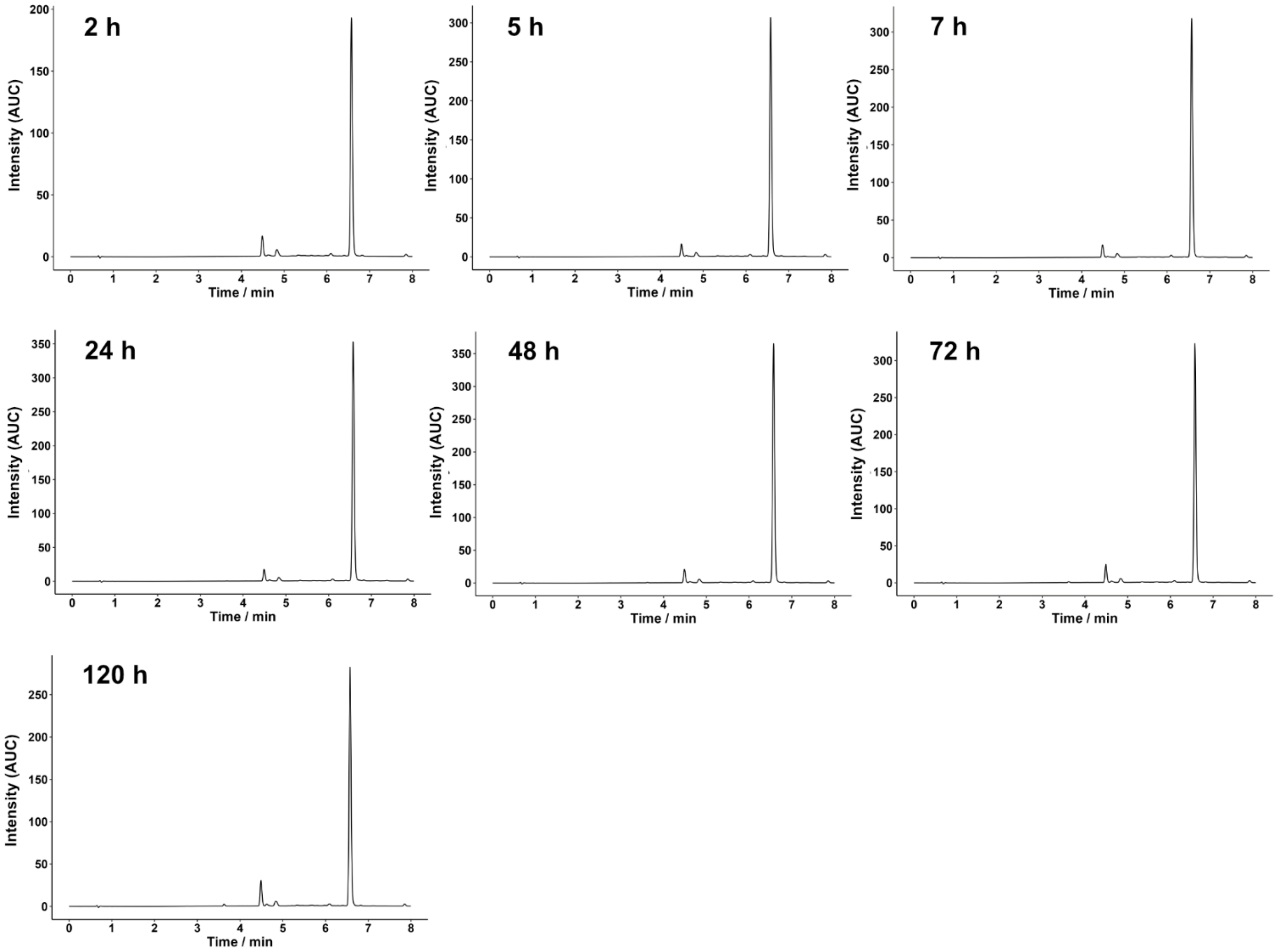

Figure S2. Stability of DBCO-Thal in water at 40 °C. The chromatograms were obtained from LC-MS. The DBCO-Thal was stored at 200 mM in DMSO, -20 °C with regular thaw-freeze cycle and kept for one and half year in the freezer after synthesis.

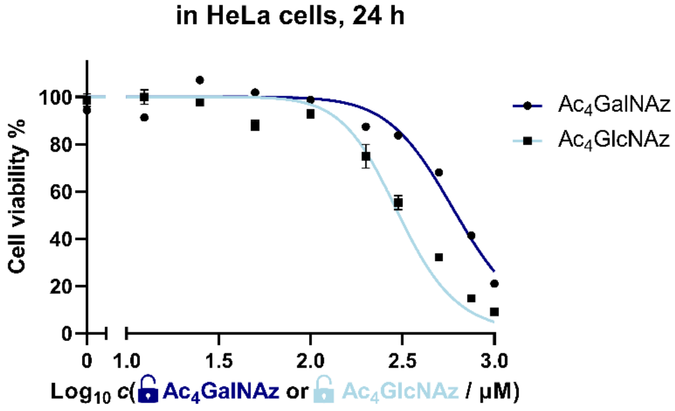

Figure S3. Cell viability assay to evaluate the cytotoxicity of Ac_4GalNAz and Ac_4GlcNAz after 24 hours incubation in HeLa cells, *n* = 3.

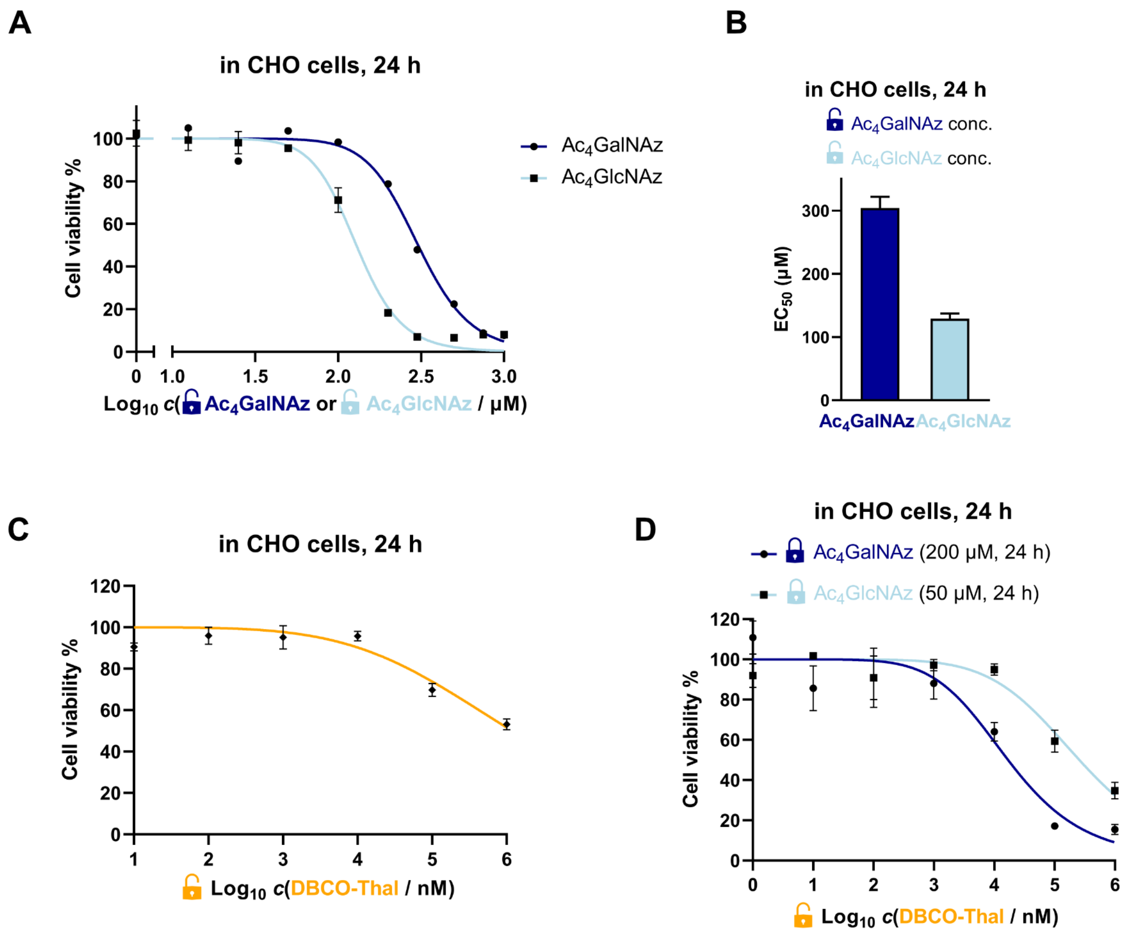

Figure S4. A) Cell viability assay to evaluate the cytotoxicity of the $Ac_4GalNAz$ and $Ac_4GlcNAz$ in CHO cells, *n* = 3. $Ac_4GalNAz$ gave EC_{50} value at 303 μM and $Ac_4GlcNAz$ at 129 μM, with no cytotoxicity up to 200 μM and 80 μM, respectively. **B**) Bar plot comparing cytotoxicity of $Ac_4GalNAc$ and $Ac_4GlcNAc$ analogues in CHO cells after 24 hours incubation, *n* = 3. **C**) Concentration-dependent cytotoxicity of DBCO-Thal in CHO cells after 24 hours incubation. Concentration range between 10 nM and 1 mM, *n* = 3. **D**) DBCO-Thal concentration-dependent cytotoxicity after incubation with $Ac_4GalNAz$ (200 μM, 24 hours) or $Ac_4GlcNAz$ (50 μM, 24 h) in CHO cells, *n* = 3. DBCO-Thal gave EC_{50} value at 17 and 257 μM for $Ac_4GalNAz$ or $Ac_4GlcNAz$ pre-treated CHO cells, respectively.

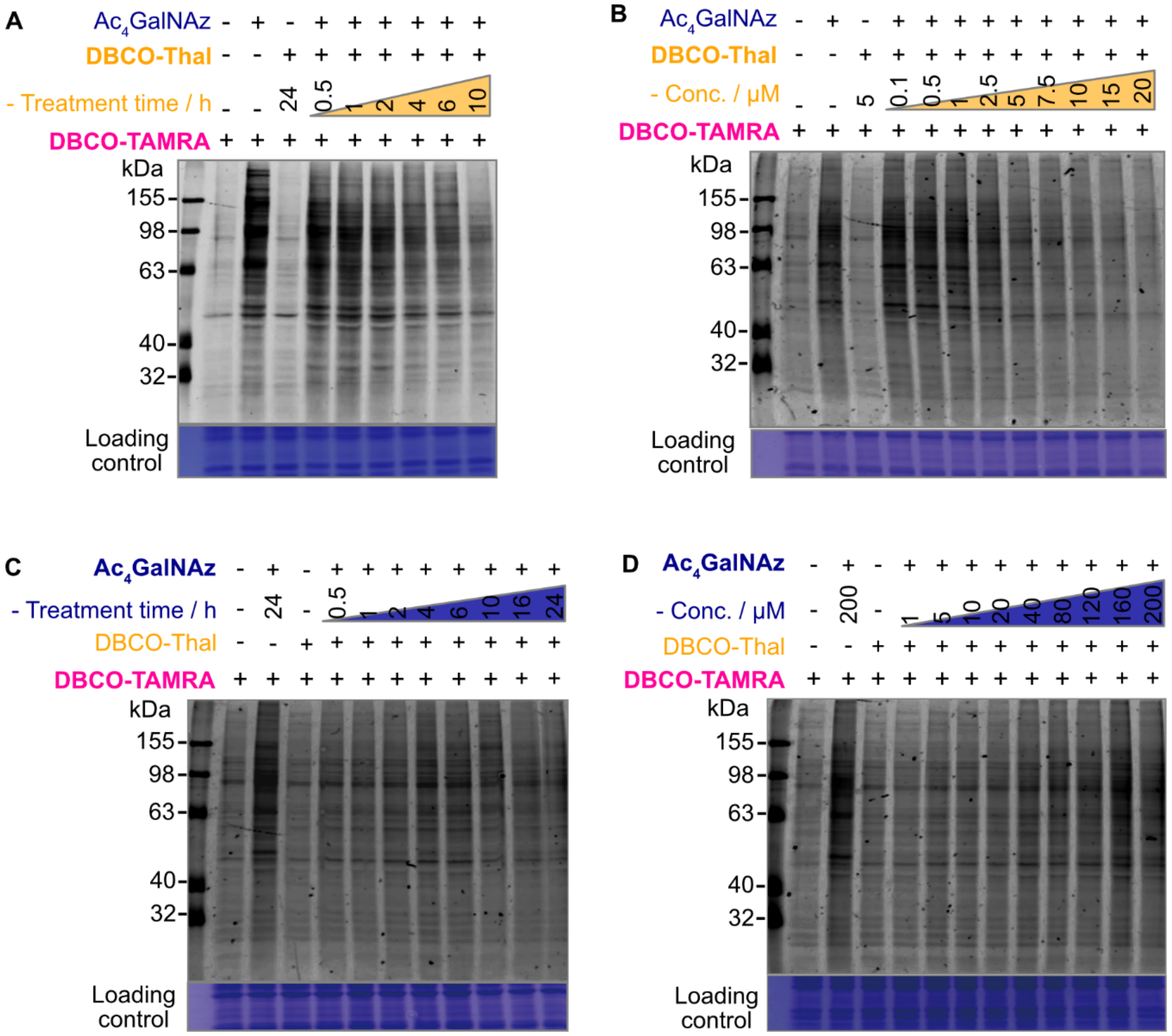

Figure S5. In-gel fluorescence analysis of cell lysates harvested after GlyTAC treatments using DBCO-TAMRA. For each sample, 20 μg proteins were incubated with 15 mM iodoacetamide for 30 minutes and then stained with 20 μM DBCO-TAMRA for 30 minutes at room temperature.

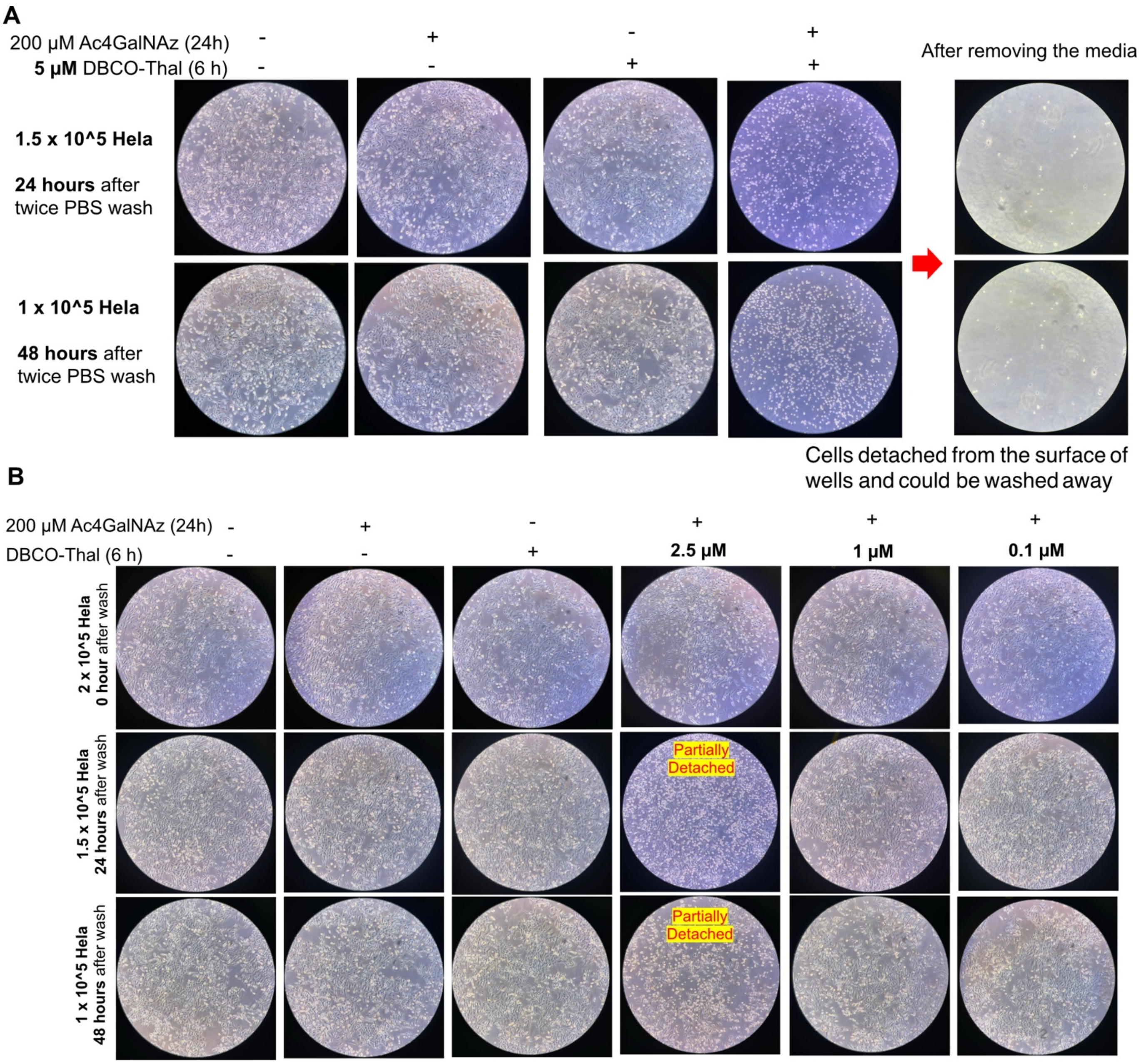

Figure S6. Persistence of GlyTAC approach in HeLa cells, with 5 µM **A**) or 2.5 – 0.1 µM DBCO-Thal **B**). The images were collected using camera mounted on brightfield inverted microscope (Leica, 11090137002).

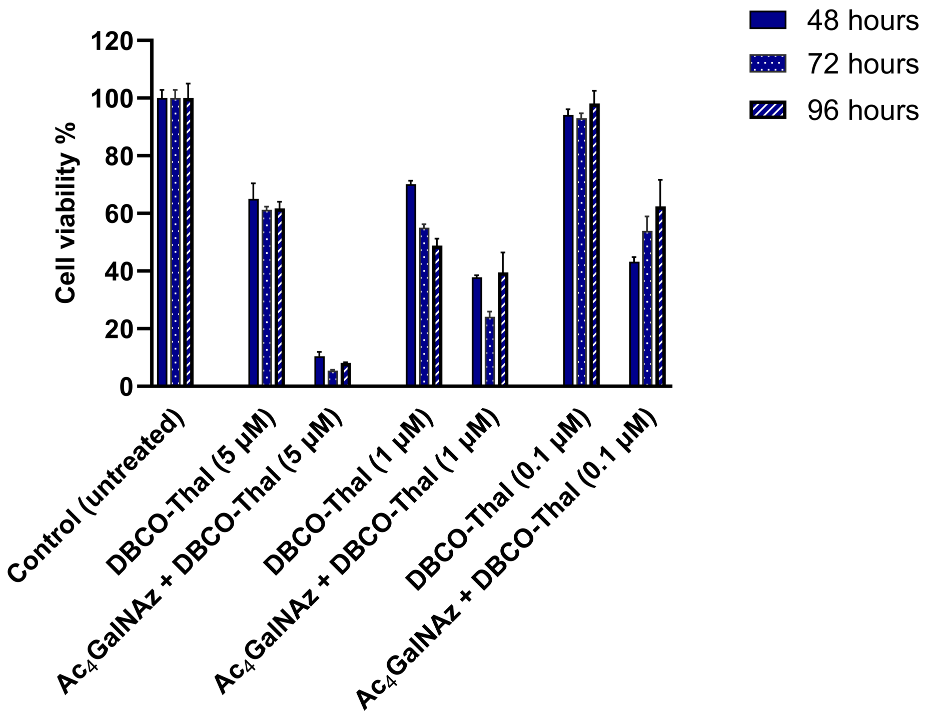

Figure S7. MTT cell viability assay evaluating the efficacy of prolonged treatment time with DBCO-Thal (5, 1 and 0.1 µM) for 48, 72 and 96 h, with $Ac_4GalNAz$ pre-treatment (200 µM, 24 h) or vehicle.

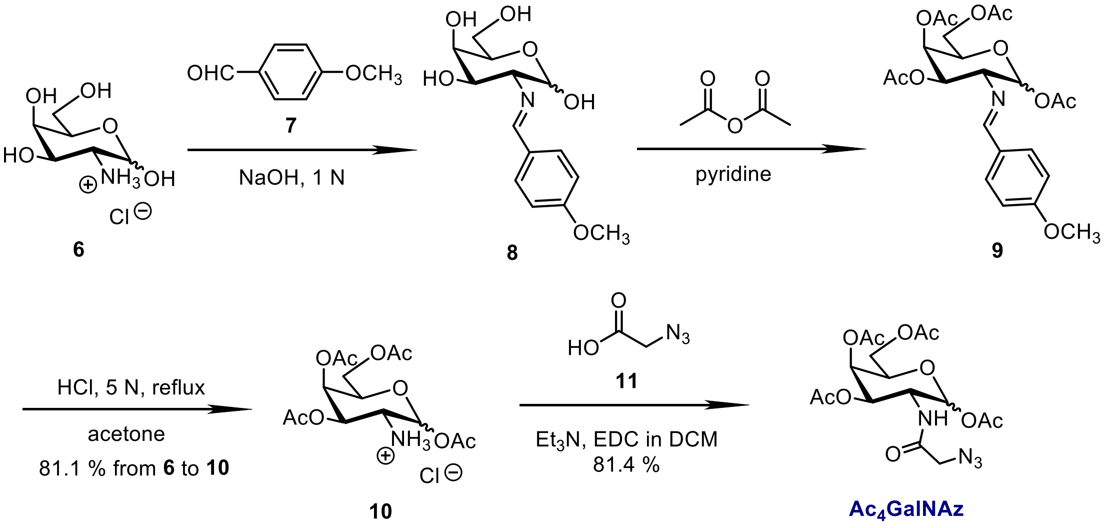

Figure S8. Synthesis of $Ac_4GalNAz$.

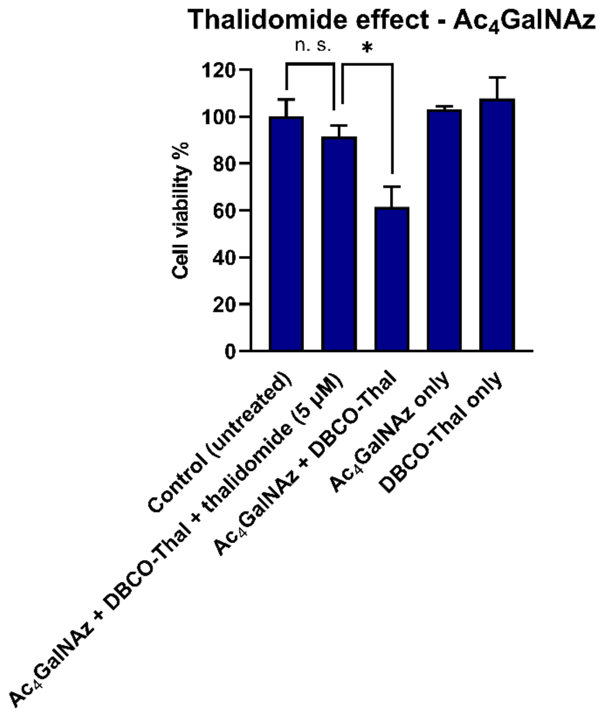

Figure S9. Thalidomide competition rescued GlyTAC-triggered cell death. The data were represented as mean values with standard deviation (n = 3) and analyzed using GraphPad Prism 9 software (GraphPad Prism 8.0.2). A p-value of < 0.05 is considered to indicate a statistically significant difference and marked with ✱ and n.s. indicates not significant statistically.

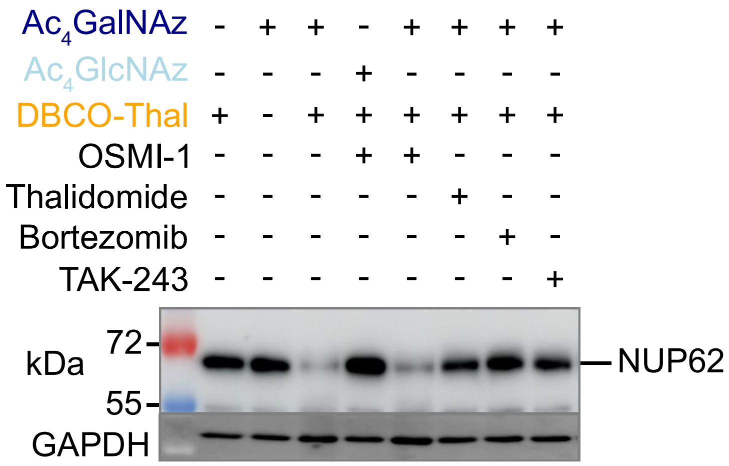

Figure S10. Western blot of NUP62 verifying the rescuing effects of OSMI-1, thalidomide, bortezomib and TAK-243.

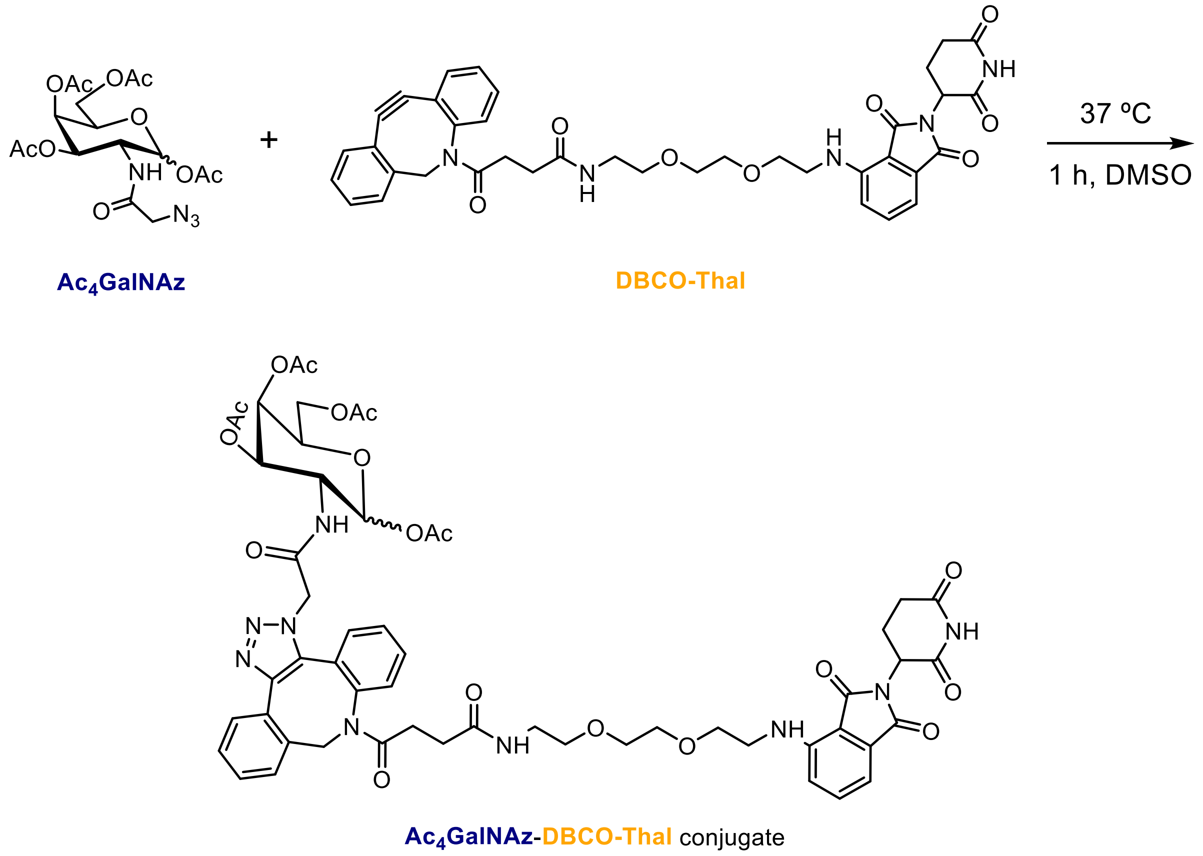

Figure S11. Synthesis of Ac$_4$GalNAz-thalidomide conjugate.

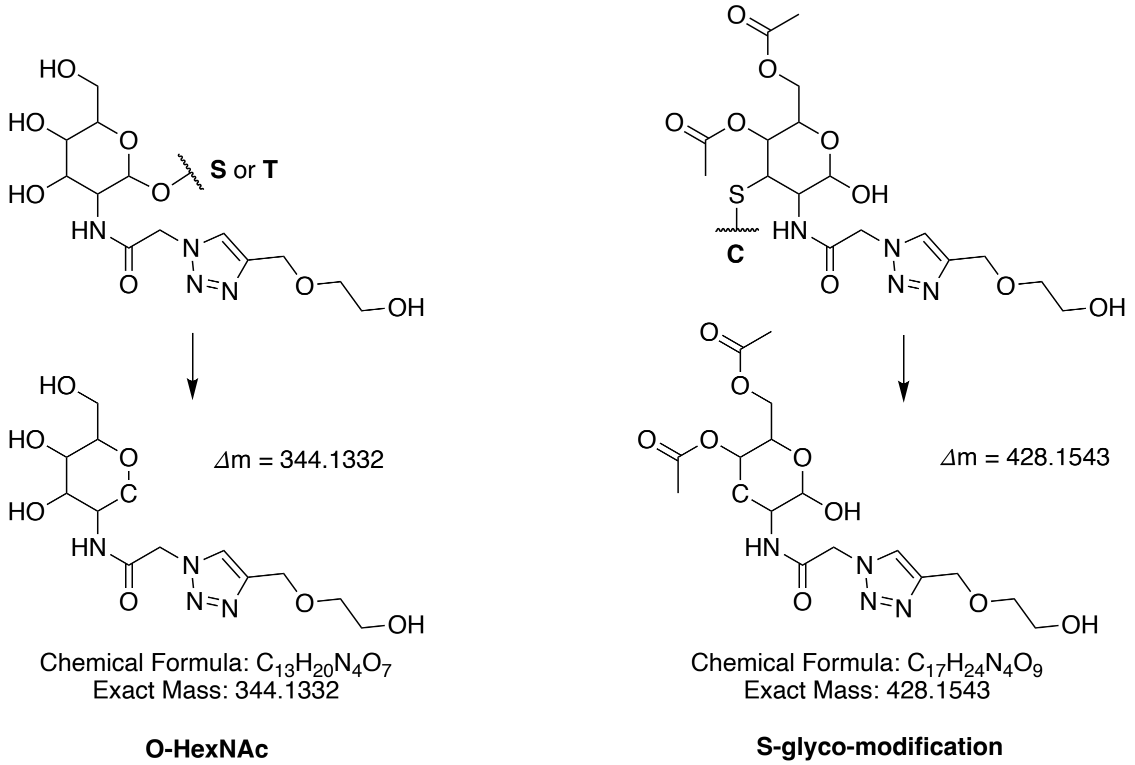

Figure S12. Structures of Ac$_4$GalNAz metabolically and chemically labelled glycopeptides. We have reanalysed the data from publication by Woo *et al.* acquired on LTQ-Orbitrap Elite (PRIDE repository PXD004559).[31] They carried out the pull-down of the *O*-linked glycopeptides from Ac$_4$GalNAz treated cells using acid-cleavable biotin-alkyne linker, which can result in glycopeptides above shown 'natural' *O*-GlcNAc and *S*-glyco-modification. The spectra (341_iso_glycan_trypsin_include.raw;

341_iso_glycan_trypsin_scout.raw; 5162_18h_iso_trypsin_glycan_include.raw; 5162_18h_iso_trypsin_glycan_scouting.raw; 341_unlabeled_glycan_C.raw and 341_unlabeled_glycan_C_include.raw) were searched by MSFragger (v19.1) closed search with 10 ppm precursor mass tolerance including variable modifications on serine and threonine (+344.1332 Da) and on cysteine (+428.1543 Da). Numbers of identified peptides with corresponding modification are summarised in **Table S1**. There is in average 300 *O*-HexNAc peptides and 66 *S*-glyco-modified peptides per sample, which results in the ratio of 4.5 : 1 of metabolic : chemical modification, in line with the conclusion from Mukherjee *et al.*.[30] Most proteins are metabolically labelled with approximately 18 % chemical *S*-glyco-modification content.

Table S1. Results of MSFragger search for metabolically and chemically labelled glycopeptides.

	# of identified glycopeptides	
**treatment**	**O-HexNAc**	***S*-glyco-modification**
Ac_4GalNAz	266	61
Ac_4GalNAz	456	92
Ac_4GalNAz	173	46
Ac_4GalNAz	308	67
control	1	0
control	0	0

Methods

Organic synthesis

Reagents and solvents were purchased from commercial suppliers, including abcr GmbH (Karlsruhe, Germany), Acros Organics (Thermo Fisher Scientific, USA), Sigma-Aldrich (St. Louis, USA), TCI Deutschland GmbH (Eschborn, Germany), Thermo Scientific (Loughborough, UK) and they were used without any further purification. Of note, *N*-azidoacetylglucosamine-tetraacylated (Ac_4GlcNAz) was obtained from Jena Bioscience GmbH (Jena, Germany; Catalog # CLK-1085-5); DBCO-PEG_4-5-tetramethylrhodamine (DBCO-PEG_4-5-TAMRA) was obtained from Jena Bioscience GmbH (Jena, Germany; Catalog # CLK-A131N-1); 3-(4,5-dimethyl-2-thiazolyl)-2,5-diphenyl-2*H*- tetrazolium bromide (MTT) was obtained from Merck KGaA (Darmstadt, Germany; Product No. # M2128); DBCO-NHS ester (**5**) was obtained from abcr GmbH (Karlsruhe, Germany; Article ID # AB549646); (+/-)-thalidomide was obtained from abcr GmbH (Karlsruhe, Germany; Article ID # AB347541); bortezomib was obtained from TCI Deutschland GmbH (Eschborn, Germany; Article ID # B5741), TAK-243 was obtained from biomol GmbH (Hamburg, Germany; Article ID # Cay30108-1). 1,3,6-tri-*O*-acetyl-4-deoxy-*N*-azidoacetyl-glucosamine ($Ac_3$4dGlcNAz) was synthesized in Prof. Dr. Anja Hoffmann-Röder's lab at LMU Munich.

TLC (thin layer chromatography) was performed to monitor the reaction progress, which was done on pre-coated silica gel plates (60 F-254, 0.25 mm, from Merck KGaA, Darmstadt, Germany) as the stationary phase, with detection by UV lights (λ = 254 and/or 366 nm).

Flash chromatography was performed by Pure Chromatography Instruments (Pure-C815 Flash) from BÜCHI Labortechnik GmbH (Essen, Germany) on pre-fabricated column (FlashPure silica gel, 40 µm irregular, 12 g) with the indicated eluent.

^{1}H and proton-decoupled ^{13}C NMR spectra for compound characterization in deuterated chloroform ($CDCl_3$) were acquired at 298K on a Bruker Avance Neo 500 spectrometer (11.7 T), using a nitrogen-cooled Prodigy BBO probe. All chemical shifts are reported in delta (δ) units in parts per million (ppm) relative to distinguished solvent signals as an internal reference. Coupling constant *J* are indicated in Hertz (Hz). Splitting patterns for peak assignments are indicated as the following abbreviations: br, broad; s, singlet; d, doublet; t, triplet; q, quartet; m, multiplet; dd, doublet of doublets; dt, doublet of triplets. Spectra were analyzed using MestReNova (version 14.2.1, Mestrelab Research, Santiago de Compostela, Spain).

LC-MS chromatograms were obtained from Thermo Fisher LC-MS system composed of a DIONEX UltiMate 3000 HPLC system (pump, auto sampler, column compartment, and diode array detector) and an ESI (electrospray ionization)-MS based MSQ Plus single quadrupole mass spectrometer; the latter one was used for ESI-MS measurements with direct injection. Reversed phase column chromatographic methods use a Hypersil Gold C18 selectivity column (100 × 2.1 mm).

DBCO-Thal *synthesis*

4-Fluorothalidomide (**1**, 100 mg, 0.365 mmol, 1.0 equiv.) was dissolved within 4 mL DMF, then mono-*N*-Boc-protected diamine (**2**, 95.3 µL, 0.402 mmol, 1.1 equiv.) and DIPEA (190.7 µL, 1.095 mmol, 3.0 equiv.) were added dropwise. The resulting mixture was continuously heated in an oil bath at 130 °C for 16 hours. Afterwards the reaction mixture was repeatedly co-evaporated with toluene and the residues were purified by flash chromatography over silica gel, eluting with EtOAc in hexane (0 - 100 %). The product, 4-(mono-*N*-Boc-diamine)thalidomide (**3**), was obtained as a bright yellowish oil with a yield at 73.9 % (136 mg).[2]

ESI-MS (m/z) calculated mass for product (**3**) $C_{24}H_{33}N_4O_8$ $[M+H]^+$: 505.23; observed mass: 505.43.

^{1}H NMR (500 MHz, $CDCl_3$) δ (ppm) 8.45 (s, 1H), 7.52 – 7.43 (m, 1H), 7.12 – 7.05 (m, 1H), 6.89 (dd, *J* = 13.8, 8.5 Hz, 1H), 6.52 (d, *J* = 7.1 Hz, 1H), 5.05 (s, 1H), 4.96 – 4.85 (m, 1H), 3.71 (q, *J* = 4.9 Hz, 2H), 3.65 (qt, *J* = 5.7, 2.9 Hz, 4H), 3.58 – 3.54 (m, 3H), 3.47 (q, *J* = 5.4 Hz, 2H), 3.32 (q, *J* = 5.7 Hz, 2H), 2.87 – 2.79 (m, 1H), 2.75 (td, *J* = 13.1, 6.4 Hz, 1H), 2.16 – 2.08 (m, 1H), 1.66 (s, 1H), 1.43 (d, *J* = 3.6 Hz, 9H).

To a solution of 4-(mono-*N*-Boc-diamine)thalidomide (**3**, 136 mg, 0.27 mmol) was added dropwise 9 mL TFA and 1 mL water. The resulting mixture was stirred at room temperature for 1 hour. Afterwards the solvents were removed by adding methanol, followed by repeatedly co-evaporation. The residues were separated by flash chromatography over silica gel, eluting with methanol in dichloromethane (0 - 10 %). The product (4-diaminethalidomide, **4**) was obtained as a yellowish solid with a yield at 95.4 % (104 mg).

ESI-MS (m/z) calculated mass for product (**4**) $C_{19}H_{25}N_4O_6$ $[M+H]^+$: 405.1774, observed mass: 405.1776.

^{1}H NMR (500 MHz, $CDCl_3$) δ (ppm) 9.66 (s, 1H), 7.47 (dd, *J* = 8.5, 7.1 Hz, 1H), 7.04 (d, *J* = 7.1 Hz, 1H), 6.86 (d, *J* = 8.5 Hz, 1H), 6.53 (s, 1H), 4.96 (dd, *J* = 11.9, 5.8 Hz, 1H), 3.84 – 3.56 (m, 8H), 3.43 (qd, *J* = 7.8, 5.2 Hz, 2H), 3.20 (s, 2H), 2.82 – 2.62 (m, 3H), 2.04 (ddd, *J* = 12.4, 6.3, 4.3 Hz, 1H), 1.35 – 1.13 (m, 1H). ^{13}C NMR (126 MHz, MeOD) δ 173.2, 170.4, 169.5, 167.8, 146.8, 135.9, 132.5, 116.8, 110.8, 109.9, 70.2, 69.9, 69.1, 66.5, 41.7, 39.4, 30.8, 22.4.

To a solution of 4-diaminethalidomide (**4**, 52 mg, 0.129 mmol, 1.1 equiv.) in 3 mL DMF was added DBCO-NHS ester (**5**, 47 mg, 0.117 mmol, 1 equiv.) and DIPEA (61.1 μL, 0.351 mmol, 3.0 equiv.), and the resulting mixture was gently stirred at room temperature for 30 minutes. Afterwards the reaction mixture was repeatedly co-evaporated with toluene and the residues were purified by flash chromatography over silica gel, eluting with methanol in dichloromethane (0 - 12 %). The product (**DBCO-Thal**) was obtained as a yellowish solid with a yield at 92.1 % (70 mg).

ESI-MS (m/z) calculated mass for product (**DBCO-Thal**) $C_{38}H_{38}N_5O_8$ $[M+H]^+$: 692.2720, observed mass: 692.2730.

^{1}H NMR (500 MHz, $CDCl_3$) δ (ppm) 7.69 (dd, *J* = 7.2, 5.0 Hz, 1H), 7.52 – 7.43 (m, 1H), 7.42 – 7.26 (m, 4H), 7.23 (ddd, *J* = 7.0, 5.4, 1.7 Hz, 1H), 7.10 (d, *J* = 7.1 Hz, 1H), 6.87 (dd, *J* = 8.5, 3.4 Hz, 1H), 6.52 (dt, *J* = 11.4, 5.5 Hz, 1H), 6.17 (dt, *J* = 39.9, 5.5 Hz, 1H), 5.16 (dd, *J* = 13.9, 9.3 Hz, 1H), 4.92 – 4.82 (m, 1H), 3.75 – 3.49 (m, 7H), 3.47 – 3.26 (m, 5H), 2.91 – 2.65 (m, 4H), 2.54 – 2.38 (m, 1H), 2.21 – 2.05 (m, 2H), 1.92 (ddt, *J* = 31.1, 16.9, 6.0 Hz, 1H) ^{13}C NMR (126 MHz, $CDCl_3$) δ 207.2, 172.1, 171.6, 169.4, 168.9, 167.7, 162.9, 151.3, 148.1, 146.7, 136.1, 132.5, 132.4, 132.3, 128.7, 128.2, 128.1, 128.0, 127.0, 125.5, 123.2, 122.4, 116.8, 114.7, 111.6, 110.3, 91.2, 70.6, 70.1, 69.8, 69.2, 55.6, 48.9, 42.2, 36.7, 31.6, 30.9, 25.4.

***Ac_4GalNAz** synthesis*

D-(+)-Galactosamine hydrochloride (**6**, 400 mg, 1.855 mmol, 1.0 equiv.) was dissolved with in 1 N NaOH (4 mL) at 0 °C, followed by the addition of *p*-anisaldehyde dropwise (**7**, 2.26 mL, 18.55 mmol, 10.0 equiv.). The resulting mixture was then stirred vigorously at room temperature until a white precipitate was formed (approx. 20 minutes), which was placed at 4 °C for 2 hours to complete precipitation. The white crystalline was filtered, washed with iced water and Et_2O, and dried under vacuum to get the product (**8**, 341.6 mg, 1.150 mmol, 62.0 %). This compound was directly dissolved in pyridine (5 mL) and acetic anhydride (1087 μL, 11.50 mmol, 10.0 equiv.) was added dropwise at 0 °C. The reaction mixture was then stirred vigorously at 0 °C for 30 minutes, slowly warmed up to room temperature for another 16 hours with gentle stirring. Afterwards the mixtures were all poured into iced water and kept at 4 °C for 6 hours. Then the precipitate was filtered, washed with cold water and vacuum-dried to get the tetraacetylated imine intermediate (**9**, 434.9 mg, 0.935 mmol, 81.3 %). This compound was then fully dissolved in acetone (5 mL) and heated to reflux. Hydrochloric acid (HCl, 5 N, 300 μL) was quickly added into the solution and the reflux was continued for another 20 minutes. The mixture was cooled down to room temperature and poured into cold Et_2O for 2 hours. The precipitate was obtained through filtration and washing with cold Et_2O, which was further dried completely under high vacuum for 8 hours to get the product tetraacetylated galactoseamine

(**10**, 263.8 mg, 0.758 mmol, 81.1 %). This compound was used for the amide coupling without any further purification.[3–5]

ESI-MS (m/z) calculated mass for product $C_{14}H_{22}NO_9$ $[M+H]^+$: 348.1294; observed mass: 348.1297.

^{1}H NMR (500 MHz, DMSO-d_6) δ (ppm) 8.77 (d, *J* = 17.9 Hz, 3H, NH_3^+), 5.90 (dd, *J* = 8.7, 2.9 Hz, 1H, H-1), 5.32 – 5.26 (m, 2H, H-3, H-4), 4.32 – 4.28 (m, 1H, H-5), 4.04 (h, *J* = 6.7, 6.3 Hz, 2H, H-6), 3.84 – 3.66 (m, 1H, H-2), 2.17 (s, 3H), 2.13 (s, 3H), 2.00 (d, *J* = 7.8 Hz, 6H). ^{13}C NMR (126 MHz, MeOD) δ 170.6, 170.3, 169.7, 168.7, 90.5, 71.7, 69.2, 65.8, 60.8, 50.1, 19.3, 19.2, 19.1, 19.0.

To a stirring solution of azidoacetic acid (**11**, 240.84 µL, 3.217 mmol, 5.0 equiv.) in anhydrous dichloromethane (5 mL) was added 1-ethyl-3-(3-dimethylaminopropyl)carbodiimide (EDC, 249.7 mg, 1.609 mmol, 2.5 equiv.) at 0 °C. This mixture was gently stirred at 0 °C for 20 minutes for active ester formation. Meanwhile a solution of tetraacetylated galactoseamine (**10**, 224 mg, 0.643 mmol, 1.0 equiv.) in anhydrous dichloromethane was mixed with anhydrous triethylamine (134.52 µL, 0.965 mmol, 1.5 equiv.) and kept stirring at 0 °C. Then the previous mixture containing azidoacetic acid and EDC was added dropwise into tetraacetylated galactoseamine solution within 5 minutes. This complete mixture was reacted firstly at 0 °C for 1 hour, then moved to room temperature for 16 hours with stirring. After that the final compound (**Ac_4GalNAz**, 225 mg, 0.523 mmol, 81.4 %) was obtained through flash chromatography by eluting with EtOAc and hexane (3:2, v/v).[6]

ESI-MS (m/z) calculated mass for product (**Ac_4GalNAz**) $C_{16}H_{22}N_4O_{10}Na$ $[M+Na]^+$: 453.1234; observed mass: 453.1198.

^{1}H NMR (500 MHz, $CDCl_3$) δ (ppm) 6.69 (d, *J* = 9.2 Hz, 1H, NH), 5.83 (d, *J* = 8.8 Hz, 1H, H-1), 5.37 (d, *J* = 3.3 Hz, 1H, H-3), 5.26 (dd, *J* = 11.2, 3.4 Hz, 1H, H-4), 4.34 (dt, *J* = 11.3, 9.0 Hz, 1H, H-2), 4.15 – 4.08 (m, 3H, H-6a, H-6b, H-5), 3.90 (s, 2H, CH_2N_3), 2.14 (s, 3H), 2.10 (s, 3H), 2.02 (s, 3H), 1.98 (s, 3H). ^{13}C NMR (126 MHz, $CDCl_3$) δ (ppm) 170.6, 170.5, 170.3, 169.6, 167.8, 92.5, 71.7, 70.0, 66.5, 61.4, 52.6, 50.0, 20.9, 20.6, 20.6.

Ac_4GalNAz-thalidomide *conjugate synthesis*

5 µL 200 mM Ac_4GalNAz in DMSO was mixed with 5 µL 200 mM DBCO-Thal in DMSO and vortexed thoroughly. The resulting mixture was then incubated at 37 ºC for 1 hour, subjected to lyophilization to get the product as a white powder without any further purification. ESI-MS (m/z) calculated mass for product $C_{54}H_{59}N_9O_{18}Na$ $[M+Na]^+$: 1144.3876, observed mass: 1144.3823.

Cell culture

The HeLa cells were cultivated in a high-glucose Dulbecco's Modified Eagle Medium (DMEM) supplemented with 10 % (v/v) heat-inactivated fetal bovine serum (FBS) and 2 % (v/v) *L*-glutamine. Cells were maintained in 10-cm cell culture dishes for adherent cells at 37 °C under constant humidity and 5 %

CO_2 concentration. The same conditions were applied for culturing HCT116, HepG2, MCF7, SH-SY5Y, and SK-MEL-28 cell lines. For A549 and CHO cell lines, the medium was exchanged to Kaighn's Modification of Ham's F-12 (F-12K) Medium supplemented with 10 % (v/v) heat-inactivated fetal bovine serum and 2 % (v/v) *L*-glutamine. For K562 cell line, the medium was exchanged to RPMI 1640 medium supplemented with 10 % (v/v) heat-inactivated fetal bovine serum. The subcultivation ratio at approx. 1:6 was applied to all cell lines.

Treatments of two-components GlyTACs in living cells

When cells were grown to approx. 70 % confluence, the old cell culture medium was removed, followed by washing with PBS buffer once. Then fresh cell culture mediums containing GlcNAc or GalNAc analogues ($Ac_4GlcNAz$, $Ac_4GalNAz$, or $Ac_34dGlcNAz$) with specified concentrations were supplemented and incubated usually for 24 hours. Afterwards the medium was removed, and cells were washed once with PBS buffer. Another portion of fresh cell culture mediums containing DBCO-Thal were added to reach specified concentrations and incubated usually for 24 hours or longer (**Figure S7**). Alternatively, for efficacy-persistence testing, after 24 hours incubation with DBCO-Thal, the cell culture medium was exchanged for DBCO-Thal free medium, and cells were incubated further for 24 or 48 hours.

Harvesting HeLa cells

HeLa cells were harvested after GlyTAC treatments. The cell culture medium was firstly removed, then the cells were washed twice with 5 mL cold phosphate-buffered saline solution (PBS). After removing PBS washing buffer, cells were scraped with 1 mL PBS buffer, transferred to a 1.5 mL tube and placed at 4°C shortly. Afterwards the cell suspension was centrifuged at 600 rpm, 4 ºC for 5 minutes. The supernatant was then carefully removed, and the rest cell pellet was left for lysates preparation.

Lysates preparation

The cell pellet was reconstituted in 200 µL PBS buffer and was sonicated with an ultrasonic rod sonicator at 1s-on / 1s-off cycles at 20 % intensity for a total time of 10 s. The solution was clarified by centrifugation at 4°C, 14000 rpm for 15 min. The cell debris was discarded, and the supernatant was then transferred to a new 1.5 mL tube and stored at -80°C until further use.

Protein concentration measurement

Protein concentration measurement was performed with Pierce™ BCA Protein Assay Kit from Thermo Scientific.

Western blot

Western blot for NUP62 visualization (Figure 3D-F and S10)

After measuring protein concentrations, 20 µg cell lysates were taken for each sample and topped up to a total volume of 16 µL with PBS buffer. 4 µL 5 × Laemmli buffer [10 % (w/v) SDS, 50 % (v/v) glycerol, 10 % (v/v) dithiothreitol, 0.5% (w/v) bromphenol blue, 315 mM Tris/HCl, pH 6.8] was added and the resulting mixture was incubated at 95 ºC for 5 minutes. Afterwards proteins were separated using 10 % SDS-PAGE gel (each well contained all 20 µg cell lysates). 3 µL Color Prestained Protein Standard (10 - 250 kDa, BioLabs, # P7719S) was added into the first well as the marker. The separated proteins were then transferred onto a PVDF membrane using a blotting-buffer-moistened (48 mM Tris, 39 mM glycine, 0.0375 % (m/v) SDS, 20 % (v/v) methanol) blotting sandwich, which was composed of one extra thick blot paper, the PVDF transfer membrane (pre-incubation for 5 min in methanol), the SDS-PAGE gel and again one extra thick blot paper. The protein transfer was subsequently carried out for 30 minutes at 25 V using a Semi-Dry Blotter (Bio-Rad). In order to block non-specific binding sites, the PVDF membrane was incubated for 60 minutes in blocking solution (0.5 g non-fat milk powder in 10 mL PBST (PBS +0.5 % Tween-20)) at room temperature. Afterwards 10 µL primary antibody against NUP62 (BD Biosciences, # 610498) was added to the blocking solution and the mixture was incubated overnight at 4 ºC. The PVDF membrane was washed 3 times for 15 minutes with PBST before 1 µL HRP-linked secondary antibody (Cell Signaling Technology, # 7076S) in 10 mL blocking solution (0.5 g non-fat milk powder in 10 mL PBST) was added. After 1 hour incubation at room temperature, the PVDF membrane was washed again 3 times for 15 minutes with PBST. Then, 400 µL ECL Substrate and 400 µL peroxide solution were mixed on-site and evenly distributed onto the PVDF membrane for staining. Finally, chemiluminescence signals from the membrane were captured by developing machine Amersham Imager 680 (GE Healthcare).

Cell treatment (Figure S10)

For GlyTAC treatment, HeLa cells were incubated firstly with 50 µM Ac_4GlcNAz or 200 µM Ac_4GalNAz for 24 hours. The cell culture medium was then exchanged with fresh medium containing 5 µM DBCO-Thal (for Ac_4GalNAz) or 100 µM DBCO-Thal (for Ac_4GlcNAz), as indicated on the figure.

Lane 4 & 5: For studying OSMI-1 effect on Ac_4GlcNAz (or Ac_4GalNAz) and DBCO-Thal treated HeLa cells, fresh medium containing 100 nM OSMI-1 was firstly incubated alone with cells for 3 hours. Then new medium containing 50 µM Ac_4GlcNAz or 200 µM Ac_4GalNAz was mixed and incubated together with 100 nM OSMI-1 for 24 hours more. The medium mixture was subsequently aspirated and new containing 5 µM DBCO-Thal (for Ac_4GalNAz) or 100 µM DBCO-Thal (for Ac_4GlcNAz) was added and incubated for exactly 24 hours.

Lane 6, 7, & 8: For studying (+/-)-thalidomide (or bortezomib, or TAK-243) effect on Ac_4GalNAz and DBCO-Thal treated HeLa cells, fresh medium containing 200 µM Ac_4GalNAz was firstly added after overnight seeding and incubated for exactly 24 hours. Afterwards the medium was aspirated and new

medium containing 5 µM (+/-)-thalidomide (or 5 nM bortezomib) was added and incubated for 2 hours. Then new medium containing 5 µM (+/-)-thalidomide (or 5 nM bortezomib) and 5 µM DBCO-Thal was added. The medium was incubated with cells for 8.5 hours. For TAK-243, the medium containing 200 µM $Ac_4GalNAz$ was aspirated and new medium containing 75 nM TAK-243 was added and incubated for exactly 3 hours. Then new medium containing 75 nM TAK-243 and 5 µM DBCO-Thal was added. The medium was incubated with cells for 22 hours.

The steps for NUP62 visualization were same as mentioned above.

Western blot for ubiquitinated protein visualization (Figure 3A-C)

The blocking solution was prepared by mixing 1 mL ROTI®Block (10 × conc., from Carl Roth GmbH) with 9 mL water. The primary antibody (Ubiquitin Polyclonal Antibody) used was purchased from Thermo Fischer (# PA1-187) and the HRP-linked secondary antibody was bought from Thermo Fischer (# 31460), which was diluted at a 1:20,000 ratio (i.e., 1 µL secondary antibody in 20 mL blocking solution). All other steps were identical with western blot for NUP62 visualization.

MTT cytotoxicity assay

General methods for cell seeding and treatments

Cell concentration was measured by mixing 10 µL cell suspension during subcultivation and 10 µL Trypan Blue (invitrogen, Catalog # T10282) with Countess 3 (invitrogen). Afterwards cells were seeded at a density of 5,000 living cells per well (100 µL from a solution of 50,000 living cells / mL) in a transparent, flat-bottom 96-well plate. Cells were grown overnight in a humidified atmosphere at 37 °C and 5 % CO_2. On the next day, the medium was exchanged with fresh medium supplemented with either DMSO (max. 1 % (v/v)) or other active compounds based on experimental purposes. The cells were incubated for a certain period of time, followed by subsequent incubation with other active compound(s) in newly-exchanged medium or direct addition of MTT solution.

General methods for MTT addition and cell viability measurements

To determine metabolic activity of cells, 20 µL MTT in sterile PBS solution (5 mg / mL) were added into each well. The resulting mixtures were pipetting up and down gently and incubated for exactly 4 hours. Then the medium was completely removed and the formazan crystals were resuspended in 200 µL DMSO, and the absorbances at both 570 nm and the reference wavelength 630 nm were determined in an infinite F200 pro plate reader (Tecan). All data points were measured in biological triplicates. The data was normalized with respect to the DMSO control.

For single compounds (Figure 1C-D, 4E-F, S3, and S4 A-C)

For measuring the cytotoxicity of $Ac_4GalNAz$, $Ac_4GlcNAz$, $Ac_34dGlcNAz$, DBCO-Thal, and $Ac_4GalNAz$-thalidomide conjugate, the fresh medium was added after overnight seeding to the listed concentrations and incubated for exactly 24 hours. Afterwards MTT solution in PBS was added and cell viability was measured. The highest, non-toxic (at least 80 % cell survival) concentration for each compound was applied for following experiments.

For continuous treatments (Figure 1E, 2A-B, 2D-F, and S4D)

For measuring the combined cytotoxicity of $Ac_4GalNAz$, $Ac_4GlcNAz$ or $Ac_34dGlcNAz$ and DBCO-Thal in either HeLa or CHO cells, the fresh medium containing $Ac_4GalNAz$, $Ac_4GlcNAz$ or $Ac_34dGlcNAz$ was firstly added after overnight seeding to the listed concentrations and incubated for exactly 24 hours. Then the medium was aspirated and new medium containing DBCO-Thal was added to the listed concentrations and incubated for exactly 24 hours. Afterwards MTT solution was added, and cell viability was measured. The EC_{50} value of DBCO-Thal was thus confirmed when fixing $Ac_4GalNAz$ or $Ac_4GlcNAz$ concentration, and vice versa. These two concentrations were then applied as “standard conditions” for the following time-dependent experiments, mechanistic studies and cancer cell line screenings.

For time-dependent experiments (Figure 2B and 2F)

For measuring half-life time ($t_{1/2}$) of HeLa cells when subjected to combined treatments of $Ac_4GalNAz$, $Ac_4GlcNAz$ or $Ac_34dGlcNAz$ and DBCO-Thal, the fresh medium containing $Ac_4GalNAz$, $Ac_4GlcNAz$ or $Ac_34dGlcNAz$ was firstly added after overnight seeding and incubated for exactly 24 hours. Then the medium was aspirated and new medium containing DBCO-Thal was added and incubated for the listed time. The concentrations used for both compounds were standard conc. determined from previous experiments. Afterwards MTT solution in PBS was added at each time point and cell viability was measured. The $t_{1/2}$ value was calculated from the plot of cell survival curves regarding the treatment time of DBCO-Thal.

For OSMI-1 inhibition experiment (Figure 4A-B)

For studying OSMI-1 effect on $Ac_4GlcNAz$ (or $Ac_4GalNAz$) and DBCO-Thal treated HeLa cells, fresh medium containing 100 nM OSMI-1 was firstly incubated alone with cells for 3 hours. Then new medium containing 50 µM $Ac_4GlcNAz$ or 200 µM $Ac_4GalNAz$ was mixed and incubated together with 100 nM OSMI-1 for 24 hours more. The medium mixture was subsequently aspirated and new medium containing standard conc. of DBCO-Thal (100 µM for $Ac_4GlcNAz$-pretreated cells and 5 µM for $Ac_4GalNAz$-pretreated cells) was added and incubated for exactly 24 hours. Afterwards MTT solution in PBS was added and cell viability was measured.[7]

For (+/-)-thalidomide / bortezomib / TAK-243 effects experiment (Figure 4C-D, and S9)

For studying (+/-)-thalidomide (or bortezomib, or TAK-243) effect on $Ac_4GalNAz$ and DBCO-Thal treated HeLa cells, fresh medium containing 200 μM $Ac_4GalNAz$ was firstly added after overnight seeding and incubated for exactly 24 hours. Afterwards the medium was aspirated and new medium containing (+/-)-thalidomide (or bortezomib) was added to the listed concentrations and incubated for exactly 1 hour. Then new medium containing 2 × listed conc. of (+/-)-thalidomide (or bortezomib) was added, followed by mixing with 2 × standard conc. of DBCO-Thal (10 μM). The resulting medium mixture was incubated with cells for 8.5 hours. Afterwards MTT solution in PBS was added and cell viability was measured.[8,9] For TAK-243, the medium containing 200 μM $Ac_4GalNAz$ was aspirated and new medium containing TAK-243 was added to the listed concentrations and incubated for exactly 3 hours. Then new medium containing 2 × listed conc. of TAK-243 (150 nM) was added, followed by mixing with 2 × standard conc. of DBCO-Thal (10 μM). The resulting medium mixture was incubated with cells for 22 hours. Afterwards MTT solution in PBS was added and cell viability was measured.[10]

For cancer cell line screening (Figure 5H)

All cell concentrations were measured by mixing 10 μL cell suspension during subcultivation and 10 μL Trypan Blue (invitrogen, Catalog # T10282) with Countess 3 (invitrogen). Afterwards cells were seeded at a density of 5,000 living cells per well (100 μL from a solution of 50,000 living cells / mL) in a transparent, flat-bottom 96-well plate. Cells were grown overnight in a humidified atmosphere at 37 °C and 5 % CO_2. On next two days, fresh medium containing standard conc. of $Ac_4GalNAz$ (200 μM) and then DBCO-Thal (5 μM) was treated with cells sequentially (24 hours incubation for each). Control experiments included incubation with medium containing DMSO-DMSO, $Ac_4GalNAz$-DMSO, and DMSO-DBCO-Thal.

For SK-MEL-28 cell line, the seeding was altered to 10,000 living cells per well.

For K562 cell line, the suspension cells were collected after seeding and 24-hours treatment of 200 μM $Ac_4GalNAz$, and then centrifuged at 300 rcf to remove supernatants. Then cells were treated with standard conc. of DBCO-Thal (5 μM). Afterwards MTT solution in PBS was added and cell viability was measured by centrifuging at 300 rcf to remove supernatants, resuspending in DMSO and then transferring the solution to the 96-well plate for measurement.

Cell lysates labelling by DBCO-TAMRA (Figure S5)

For each sample, cell lysates containing 20 μg proteins were aspirated from stock solution. The cell lysates were then supplemented with 0.2 % SDS in PBS buffer to a total volume of 19 μL. Afterwards 1 μL 300 mM iodoacetamide in water (final concentration at 15 mM) was added to each sample and incubated at room temperature for 30 minutes in the darkness. The resulting mixture was added with approx. 0.4 mM DBCO-TAMRA in DMSO to reach the final concentration at 20 μM. The labelling was carried out also at

room temperature for 30 minutes in the darkness.

In-gel fluorescence analysis

After incubation, the reaction mixture was mixed with 5 µL 5 × Laemmli buffer (same component with the buffer used in western blotting) and boiled at 95 °C for 5 minutes. After cooling down, the mixtures in each sample were all loaded into the well within the stacking layer. The resolution of proteins was made by SDS-PAGE using 10 % acrylamide gels. BenchMark™ Fluorescent Protein Standard (invitrogen, Catalog # LC5928) was used as a maker. Afterwards the gel was scanned and visualized on Amersham Imager 680 (GE Healthcare). The loading control was obtained by Coomassie staining and then destained within 20 % methanol and 10 % acetic acid in water.

Direct injection for high-resolution MS1 analysis

High-resolution MS measurements were performed on an Orbitrap Eclipse Tribrid Mass Spectrometer (Thermo Fisher Scientific) coupled to an UltiMate 3000 Nano-HPLC (Thermo Fisher Scientific) via an EASY-Spray source (Thermo Fisher Scientific) and FAIMS interface (Thermo Fisher Scientific).

The fully lyophilized reaction mixtures from Ac_4GalNAz or Ac_4GalNAz-thalidomide conjugate were dissolved within 0.5 % FA in acetonitrile / water mixture (1:1, v/v) to a final concentration at around 10 µM. The samples were then collected by a syringe (500 µL, Hamilton) and loaded onto the pump (chemyx). To start, the flow rate for injection was set at 10 µL / min; the resolution of Orbitrap was selected between 30,000 to 240,000; the Automatic Gain Control (AGC) was 100 %, while the maximal injection time was 100 ms and the micro-scan was varied between 1-5 depending on the measurement situations. The mass window was selected according to the the molecular weight of analytes. When the compound signal appears, the flow rate was immediately decreased to 4 µL / min. The compensation voltage (CV) was scanned and chosen to achieve the highest intensity.

Cell lysates clean-up with automated SP3 protocol

The procedure was performed in a 96-well plate. Each conditions contained 3 biological replicates. 20 µL carboxylate-coated magnetic beads (1:1 mixture of hydrophilic and hydrophobic beads) were washed manually thrice with 100 µL MS-scale water and the last washing solution was kept within the 96-well plate. On the other hand, 20 µg of cell lysates were placed within the 96-well plate and diluted to 50 µL with PBS buffer. The plate was then transferred to Hamilton MicroLab PREP robot, and the last washing solution was aspirated, followed by addition of 50 µL cell lysates onto the beads. The mixture was shaked for 1 minute at 850 rpm, room temperature. Afterwards 60 µL absolute ethanol was added to each sample and incubated for 5 minutes at 850 rpm, room temperature. The supernatants were removed and the protein-bound beads were washed sequentially with 100 µL 80 % EtOH in water for three times and 100 µL acetonitrile once. An incubation for 1 minute at 850 rpm, room temperature after each addition was carried out. After removal of the last washing solution, the beads were supplemented with 100 µL 100 mM ammonium bicarbonate buffer and then digested overnight with 1 µL sequencing-grade trypsin (0.5 mg /

mL, Promega) at 37 °C, 600 rpm. The caps for each well were all carefully encapsulated to prevent the evaporation of aqueous medium. On the next day, the peptide mixtures were all transferred to the new Eppendorf tubes and the beads were washed with 50 µL and 30 µL 1% FA in water. The beads with washing solutions were incubated at 40 °C, 850 rpm for 5 minutes. The washing solutions were collected all together with digested peptide mixtures, and placed within a MS-vial. For LC-MS/MS measurement, 5 µL solutions were injected for each sample.

LC-MS/MS measurement

MS measurements were performed on Orbitrap Eclipse Tribrid Mass Spectrometer coupled to an UltiMate 3000 Nano-HPLC via a Nanospray Flex and FAIMS interface (all from Thermo Fisher Scientific). First, peptides were loaded on an Acclaim PepMap 100 µ-precolumn cartridge (5 µm, 100 Å; 300 µM ID x 5 mm, Thermo Fisher Scientific). Then, peptides were separated at 40 °C on a PicoTip emitter (noncoated, 15 cm, 75 µm ID, 8 µm tip, New Objective) that was in-house packed with ReprosilPur 120 C18-AQ material (1.9 µm, 150 Å, Dr. A. Maisch GmbH). The LC buffers were consisted of MS-grade water (A) and acetonitrile (B), both supplemented with 0.1% formic acid. The gradient was run from 4 - 35.2% B during a 60 min method (0-5 min 4%, 5-6 min to 7%, 7-36 min to 24.8%, 37-41 min to 35.2%, 42-46 min 80%, 47-60 min 4%) at a flow rate of 300 nL/min.

Data-independent acquisition (DIA)

FAIMS was performed with one CV at -45 V. One DIA cycle comprised one MS1 scan followed by 30 MS2 scans. The mass spectrometer was operated in DIA mode with following settings: Polarity: positive; MS1 Orbitrap resolution: 60k; MS1 AGC target: standard; MS1 maximum injection time: 50 ms; MS1 scan range: m/z 200-1800; RF Lens: 30 %; Precursor Mass Range: m/z 500-740; isolation window: m/z 4; window overlap: m/z 2; MS2 Orbitrap resolution: 30 k; MS2 AGC target: 200 %; MS2 maximum injection time: auto; HCD collision energy: 35 %; RF Lens: 30 %; MS2 scan range: auto.

Computation of measured mass spectra using DIA-NN

First, measured *.raw files were converted to *.mzML format with “MSConvert” from the “ProteoWizard” software package (http://www.proteowizard.org/download.html) with following settings: “peakPicking” filter with “vendor msLevel = 1”; “Demultiplex” filter with parameters “Overlap Only” and “mass error” set to 10 ppm.[11] Subsequently, they were analyzed with DIA-NN 1.8.1 and peptides were searched against Uniprot database for Homo Sapiens with included contaminants and decoys.[12] The DIA-NN settings were as follows: FASTA digest for library-free search/library generation: enabled; Deep learning-based spectra, RTs and IMs prediction: enabled; protease: trypsin; missed cleavages: 2; maximum number of variable modifications: 2; enabled modifications: *N*-term M excision, oxidation (M) and *N*-term acetylation; peptide length range: 7 - 30; precursor charge range: 2-7; precursor range: m/z 500-740; fragment ion range: m/z 200-1800; precursor FDR: 1 %; match between runs (MBR): enabled; library generation: smart profiling; quantification strategy: Robust LC; mass accuracy: 0; scan windows: 0.

Data visualization – heatmap (Figure 5B)

For statistical analysis, the generated "report.gg_matrix.tsv" table was loaded in Perseus 1.6.14.0.[13] Then, quantified values were assigned with conditions during GlyTAC treatment. Each biological replicate had the same name to constitute one group. Samples not treated with any of the active components or treated with only one of the two components were always applied as controls. Subsequently, the groups were filtered for at least two valid values out of three columns in each group. The matrix was then processed through multiple-sample test with ANOVA-significant. The rest values were $\log_2$-transformed and missing values were replaced by a normal distribution. The rows in matrix were then filtered with positive ANOVA-significant mode and further proceeded with Z-score. The hierarchical clusterings were generated with Euclidean distance and average linkage for both row and column trees, while the row clusters were defined to be 8 to classify groups of interests. The first two groups containing most proteins were therefore mainly down-regulated and up-regulated proteins after sequential GlyTAC treatment. The gene and protein lists for these groups from different GlyTAC treatment conditions are recorded separately for Venn diagram and KEGG pathway analysis.

Data visualization – volcano plot (Figure 5D)

For statistical analysis, the generated "report.gg_matrix.tsv" table was loaded in Perseus 1.6.14.0.[13] Then, quantified values were assigned with conditions during GlyTAC treatment. Each biological replicate had the same name to constitute one group. Subsequently, the groups were filtered for at least two valid values out of three columns in each group. The rest values were $\log_2$-transformed and missing values were replaced by a normal distribution. $-\log_{10}$(p-values) were obtained by a two-sided one sample Student's t-test over replicates with the initial significance level $\alpha = 0.05$ adjustment by the multiple testing correction method of Benjamini and Hochberg (FDR = 0.05) using the volcano plot function. Finally, the volcano plot values from Perseus were transferred to R and visualized properly.

Data visualization – Venn diagrams (Figure 5C and 5E)

For Figure 5C, the down-regulated proteins' lists after hierarchical clustering (5 μM and 20 μM DBCO-Thal treatment respectively) were overlapped through the Venn diagram online tool https://bioinfogp.cnb.csic.es/tools/venny/ to determine proteins numbers of each group. The results were then visualized by Affinity Designer 2.

For Figure 5E, the down-regulated proteins' lists were overlapped with downloaded human *O*-GlcNAcylation protein database https://www.oglcnac.mcw.edu/download/ (generated on 16.07.2024) also using Venn diagram online tool https://bioinfogp.cnb.csic.es/tools/venny/. The results were then visualized by Affinity Designer 2.

Data visualization – KEGG pathway analysis (Figure 5F and 5G)

The up- and down-regulated proteins after sequential GlyTAC treatment were transferred to the online tool ShinyGO 0.80 http://bioinformatics.sdstate.edu/go/ for KEGG pathway analysis. The parameters were set as default and the results were visualized directly from the website.

Uncropped images of western blot

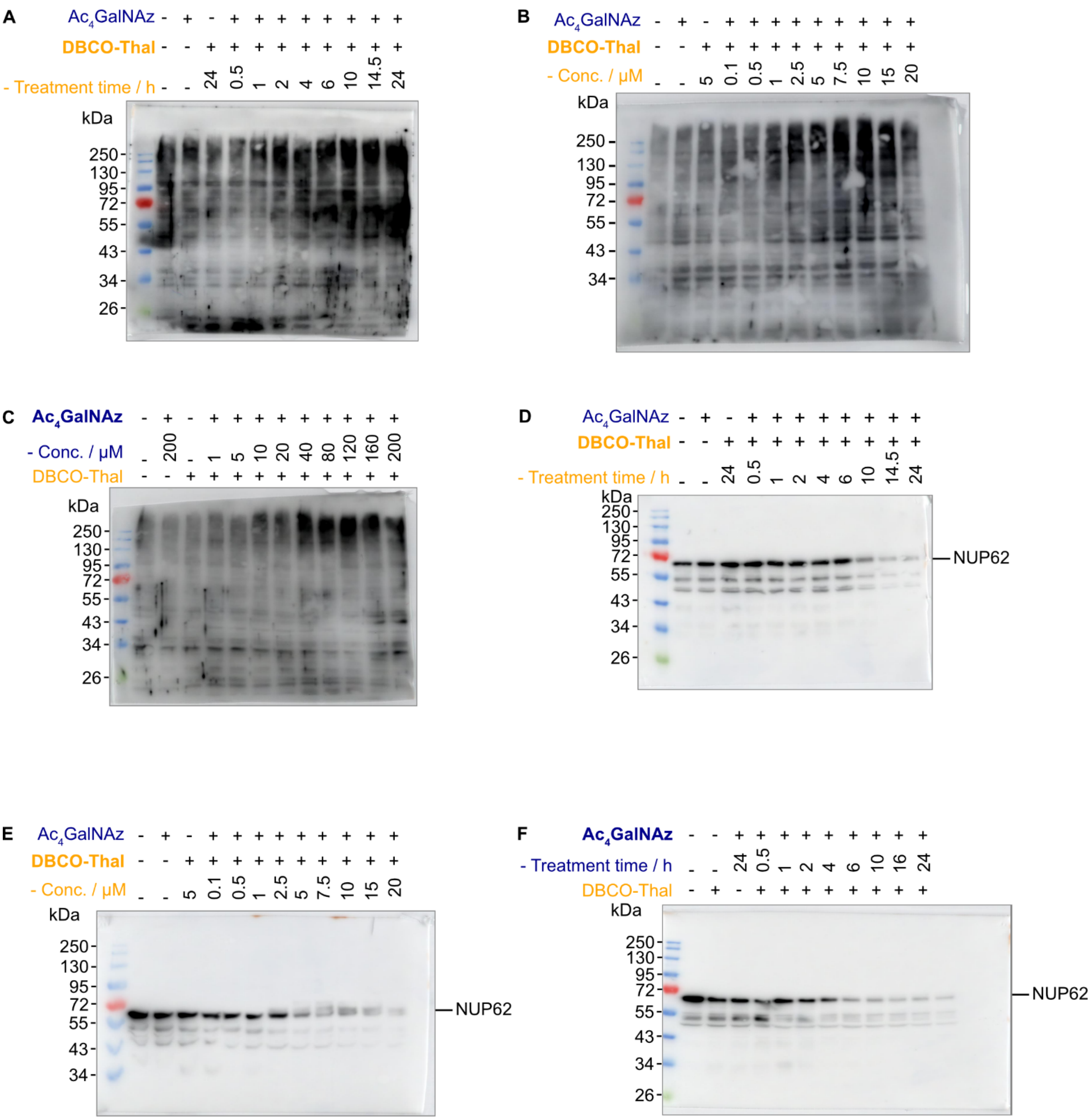

Uncropped images of **Figure 3**

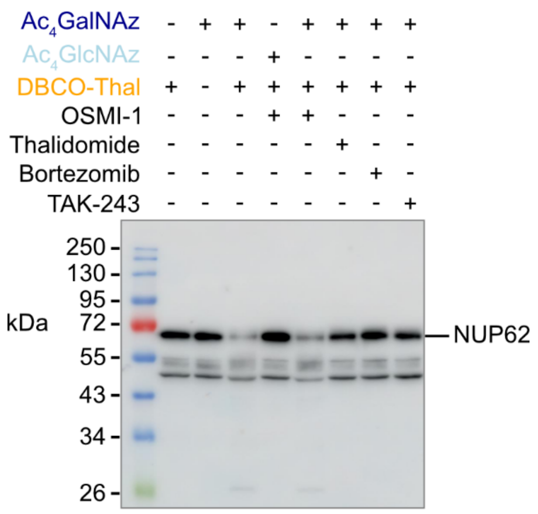

Uncropped image of **Figure S10**

NMR spectra

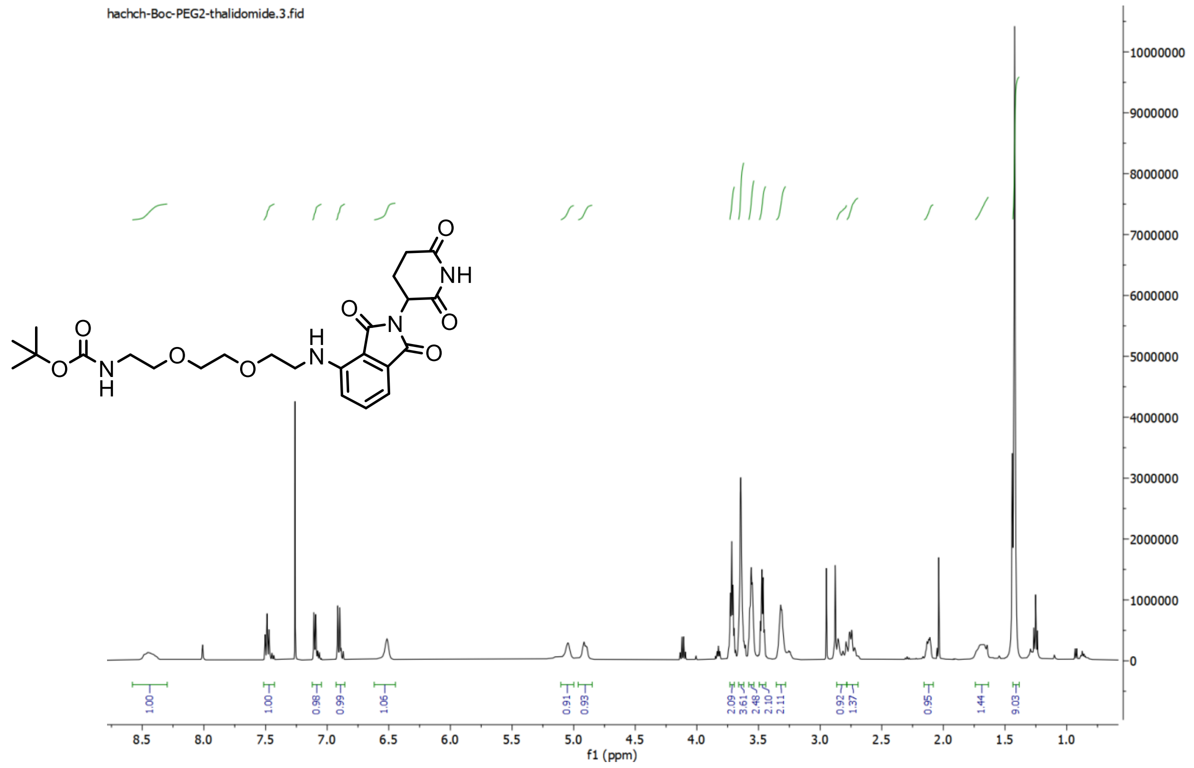

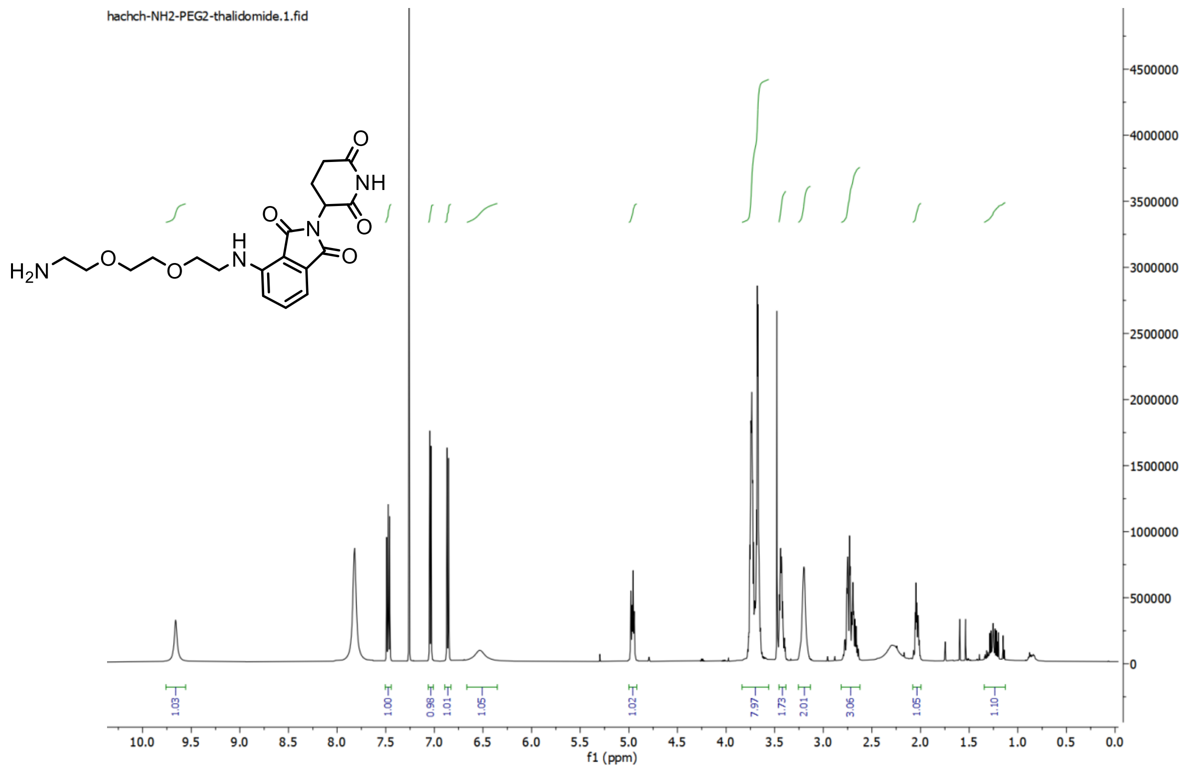
hachch-NH2-PEG2-thalidomide.1.fid
f1 (ppm)

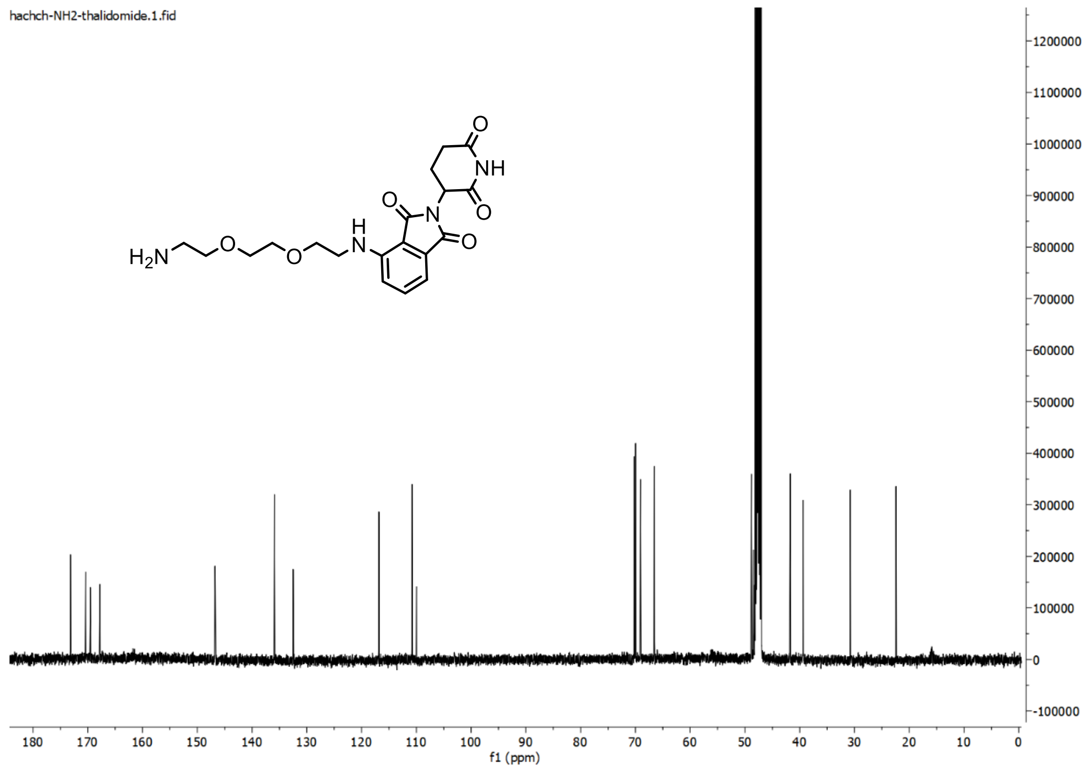
hachch-NH2-thalidomide.1.fid
f1 (ppm)

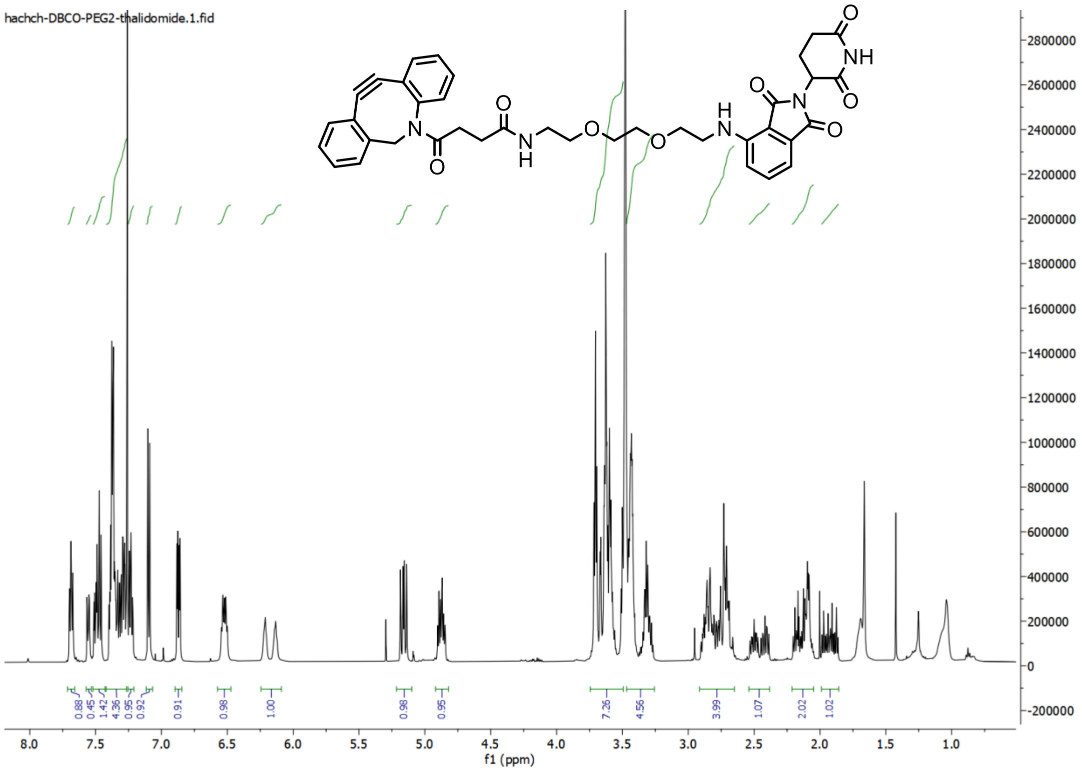
hachch-DBCO-PEG2-thalidomide.1.fid
f1 (ppm)

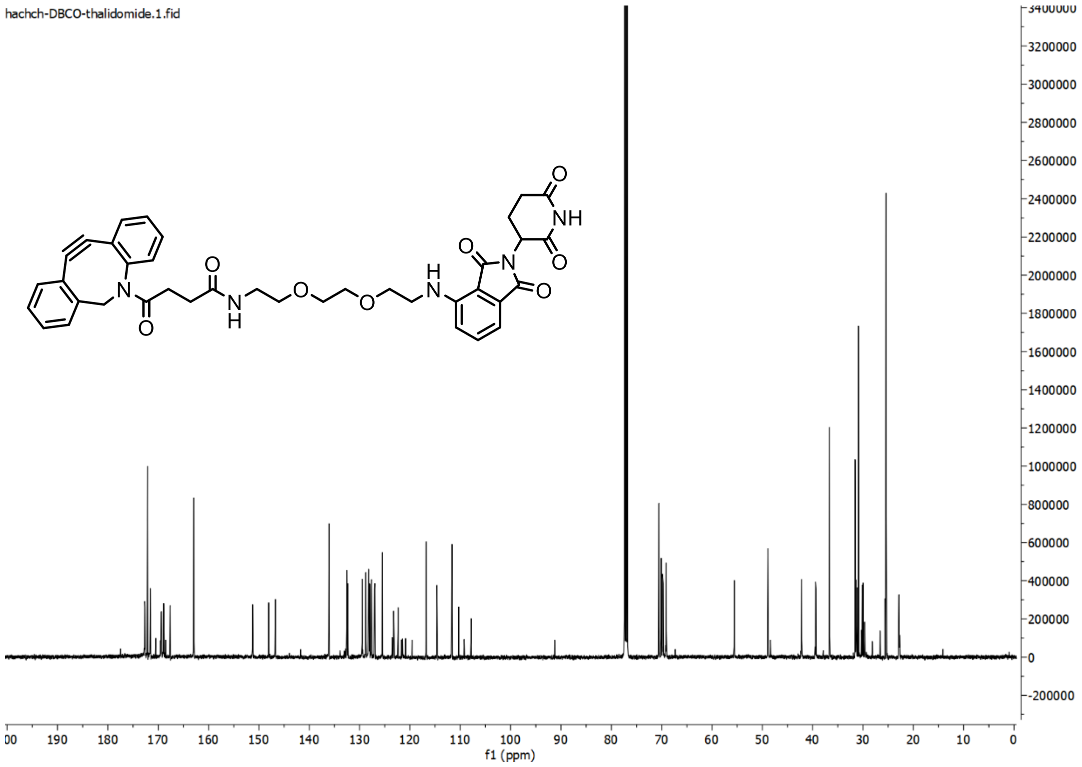
hachch-DBCO-thalidomide.1.fid
f1 (ppm)

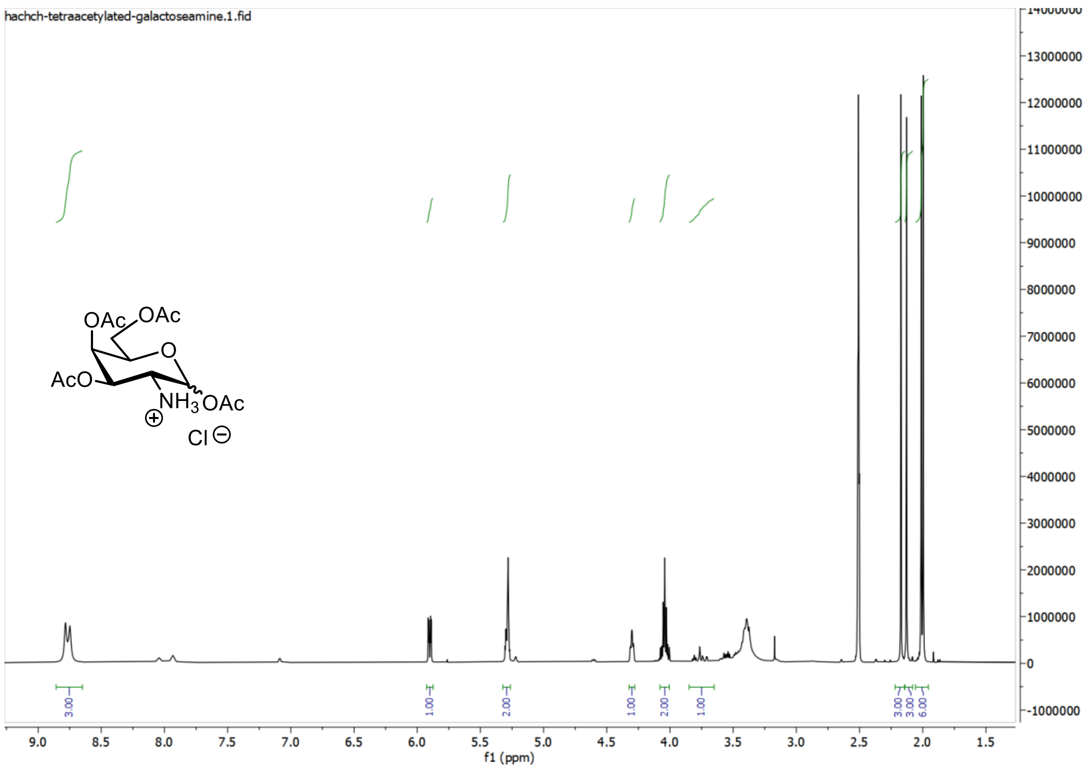
hachch-tetraacetylated-galactoseamine.1.fid
OAc
OAc
O
AcO
NH3
OAc
Cl
f1 (ppm)

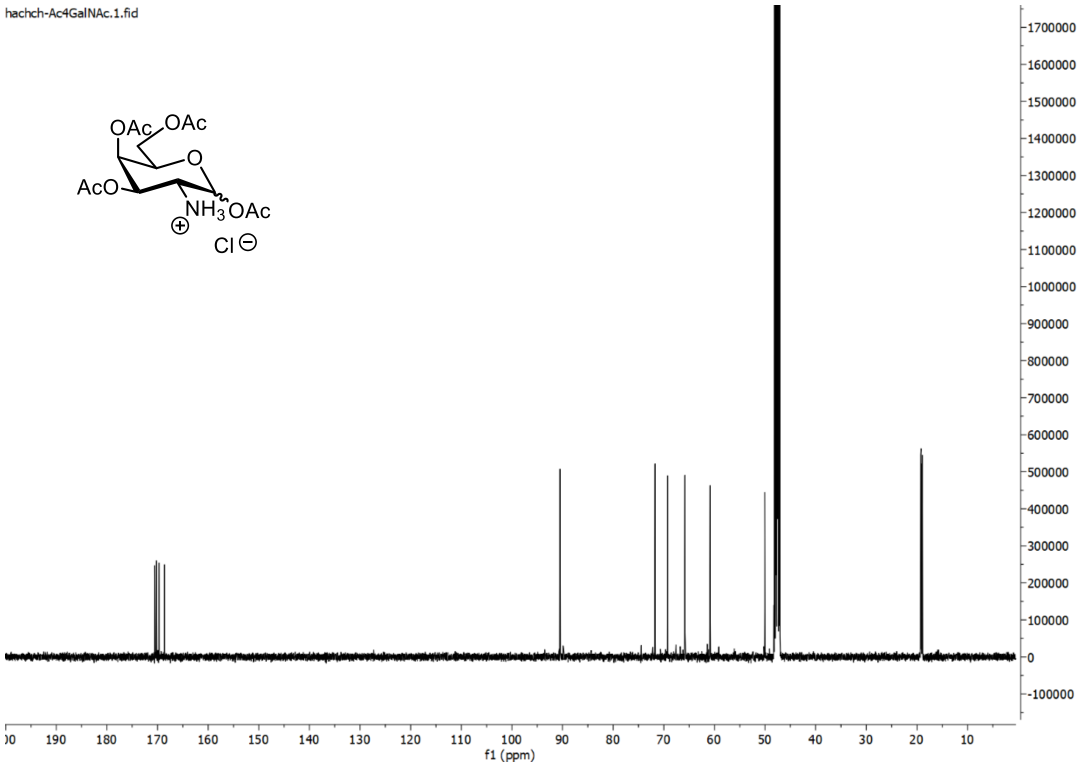
hachch-Ac4GalNAc.1.fid
OAc
OAc
O
AcO
NH3
OAc
Cl
f1 (ppm)

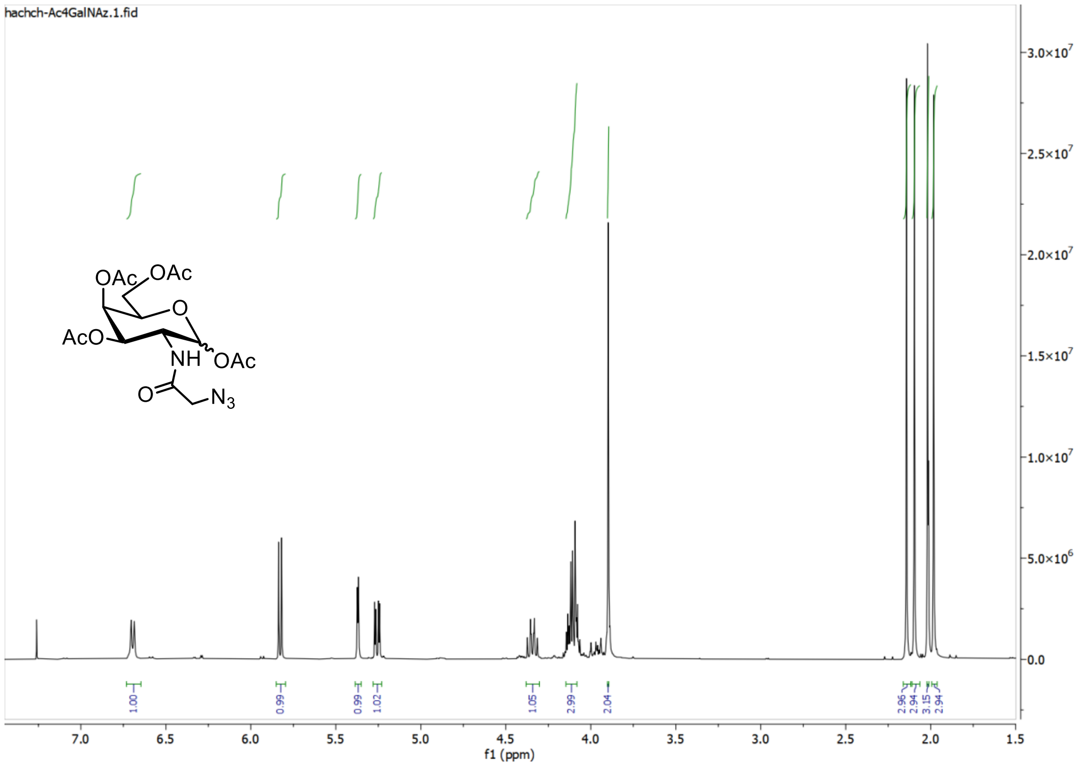
hachch-Ac4GalNAz.1.fid
OAc
OAc
O
AcO
NH
OAc
O
N3
1.00
0.99
0.99
1.02
1.05
2.99
2.04
2.96
2.94
3.15
2.94
f1 (ppm)

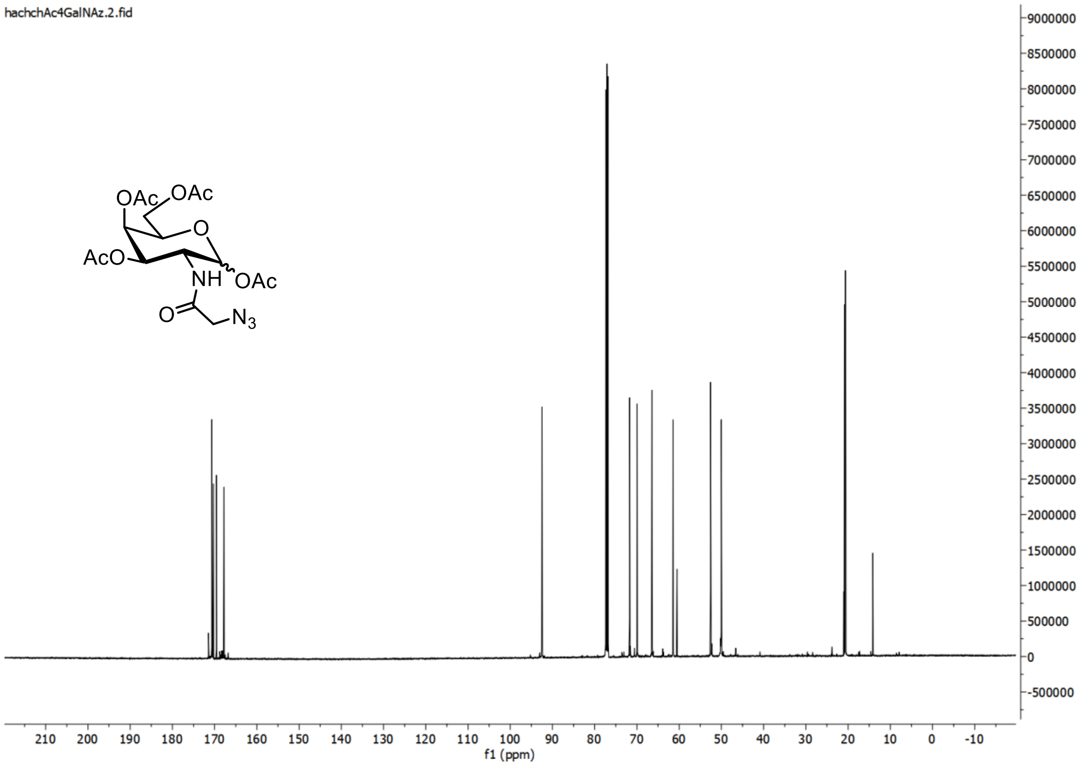
hachchAc4GalNAz.2.fid
OAc
OAc
O
AcO
NH
OAc
O
N3
f1 (ppm)

References

1. Wojdyla, K. & Rogowska-Wrzesinska, A. Differential alkylation-based redox proteomics - Lessons learnt. *Redox Biol.* **6**, 240–252 (2015).

2. Brownsey, D. K., Rowley, B. C., Gorobets, E., Gelfand, B. S. & Derksen, D. J. Rapid synthesis of pomalidomide-conjugates for the development of protein degrader libraries. *Chem. Sci.* **12**, 4519–4525 (2021).

3. Carmona, J. A. *et al.* Asymmetric organocatalytic synthesis of tertiary azomethyl alcohols: key intermediates towards azoxy compounds and α-hydroxy-β-amino esters. *Org. Biomol. Chem.* **15**, 2993–3005 (2017).

4. Agarwal, K. *et al.* Inhibition of Mucin-type O-glycosylation through metabolic processing and incorporation of N-thioglycolyl-d-galactosamine peracetate (Ac 5GalNTGc). *J. Am. Chem. Soc.* **135**, 14189–14197 (2013).

5. Metier, C. *et al.* Profiling of Haemophilus influenzae strain R2866 with carbohydrate-based covalent probes. *Org. Biomol. Chem.* **19**, 476–485 (2021).

6. Tomaszewska, J., Kowalska, K. & Koroniak-Szejn, K. Glucosamine- and galactosamine- based monosaccharides with highly fluorinated motifs. *J. Fluor. Chem.* **191**, 1–13 (2016).

7. Ortiz-Meoz, R. F. *et al.* A Small Molecule That Inhibits OGT Activity in Cells. *ACS Chem. Biol.* **10**, 1392–1397 (2015).

8. Curran, M. P. & McKeage, K. Bortezomib: A review of its use in patients with multiple myeloma. *Drugs* **69**, 859–888 (2009).

9. Sinatra, L. *et al.* Solid-Phase Synthesis of Cereblon-Recruiting Selective Histone Deacetylase 6 Degraders (HDAC6 PROTACs) with Antileukemic Activity. *J. Med. Chem.* **65**, 16860–16878 (2022).

10. Hyer, M. L. *et al.* A small-molecule inhibitor of the ubiquitin activating enzyme for cancer treatment. *Nat. Med.* **24**, 186–193 (2018).

11. Chambers, M. C. *et al.* A cross-platform toolkit for mass spectrometry and proteomics. *Nat. Biotechnol.* **30**, 918–920 (2012).

12. Demichev, V., Messner, C. B., Vernardis, S. I., Lilley, K. S. & Ralser, M. DIA-NN: neural networks and interference correction enable deep proteome coverage in high throughput. *Nat. Methods* **17**, 41–44 (2020).

13. Tyanova, S. *et al.* The Perseus computational platform for comprehensive analysis of (prote)omics data. *Nat. Methods* **13**, 731–740 (2016).
